# Thermostable Bioluminescent Intercalating Dyes for Real‐Time, Integrated Nucleic Acid Amplification and Detection

**DOI:** 10.1002/anie.6796450

**Published:** 2026-05-21

**Authors:** Yosta de Stigter, Harmen J. van der Veer, Sterre de Lignie, Robbert J. de Haas, Renko de Vries, Joost P. H. Schoeber, Anne J. M. Loonen, Adriaan J. C. van den Brule, Maarten Merkx

**Affiliations:** ^1^ Laboratory of Chemical Biology Department of Biomedical Engineering Eindhoven University of Technology Eindhoven the Netherlands; ^2^ Institute For Complex Molecular Systems Eindhoven University of Technology Eindhoven the Netherlands; ^3^ Research Group Applied Natural Sciences Fontys University of Applied Sciences Eindhoven the Netherlands; ^4^ Department of Physical Chemistry and Soft Matter Wageningen University and Research Wageningen the Netherlands; ^5^ Pathologie‐DNA Lab For Molecular Diagnostics Location Jeroen Bosch Hospital ’s‐Hertogenbosch the Netherlands

**Keywords:** bioluminescence, biosensors, nucleic acids, point‐of‐care testing, protein engineering

## Abstract

Integrated nucleic acid amplification and detection methods that can be conducted at the point of care are critical to advancing infectious disease diagnostics. We previously developed luciferase‐intercalating dye conjugates (“LUMIDs”) that allow for direct detection of double‐stranded DNA (dsDNA) through a simple, ratiometric bioluminescent readout. However, their limited thermostability hampers integration with isothermal nucleic acid amplification methods that provide the specificity and sensitivity required for diagnostics. In this work, we re‐engineered LUMID to allow for one‐pot integration with loop‐mediated isothermal amplification (LAMP) and real‐time monitoring of DNA amplification. Luciferase activity and DNA binding were retained at LAMP temperatures (60°C–65°C) by employing a thermostable NanoLuc luciferase variant and an additional thermostable DNA binding protein. To enable continuous monitoring, we additionally introduced a caged luciferin substrate with esterase‐controlled release kinetics for sustained light output. These combined advances enabled one‐pot LAMP‐LUMID assays to detect cancer‐associated human papillomavirus (HPV) subtypes with attomolar sensitivity and within ∼ 30 min. Using a simple digital camera as readout, LAMP‐LUMID demonstrated robust assay performance in patient samples, showcasing its potential as a rapid and sensitive platform for molecular diagnostics that is particularly attractive for low‐resource settings.

## Graphical abstract

We present a bioluminescent diagnostic platform that integrates DNA amplification and detection in a single step. The engineering of thermostable luciferase‐intercalating dye conjugates and controlled release of caged luciferin substrates enable real‐time, detection of attomolar concentrations of viral DNA within 30 min. This simple, camera‐readable assay offers a promising route toward rapid, point‐of‐care diagnostics, especially in resource‐limited settings.

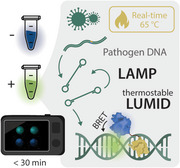

## 1 Introduction

Molecular amplification and detection of nucleic acids are essential for infectious disease diagnostics [1, 2, 3]. Quantitative polymerase chain reaction (qPCR) allows for sequence‐specific amplification of target DNA and real‐time detection using fluorescent intercalating dyes that exhibit a large increase in fluorescence upon binding non‐specifically to double‐stranded DNA (dsDNA) [4]. Although qPCR is a well‐established tool for nucleic acid diagnostics, it requires expensive equipment and expert technicians that restrict its use to centralized laboratories. The resulting long sample‐to‐answer time and the limited access to qPCR in low‐resource settings have stimulated the development of simple and rapid, isothermal nucleic acid tests that can be used at the point of need [5, 6, 7].

Isothermal nucleic acid amplification techniques (iNAATs) differ from PCR by enabling amplification at a constant temperature, eliminating the need for a thermocycler [8]. Recombinase polymerase amplification (RPA) and Loop‐mediated isothermal AMPlification (LAMP) are among the most widely used methods for DNA detection with established assays for many viral, bacterial, and parasitic targets, such as sexually transmitted infections (e.g., HPV and Chlamydia trachomatis) and tropical diseases (e.g., malaria parasite) [9, 10]. RPA is favored for its low reaction temperature (∼ 40°C), which facilitates integration of sensor components and enables simplified readout modalities with minimal heating requirements, for example, through the use of body heat [11, 12]. However, the low temperature also leads to spurious amplification, which necessitates the use of a highly target‐specific readout, such as CRISPR‐based sensors [13, 14, 15, 16, 17].

LAMP is inherently more specific owing to higher reaction temperatures (∼ 65°C) and multiple primers (4–6). Consequently, it can be integrated with simple non‐specific readouts, either colorimetric or fluorescent, suitable for point‐of‐need applications [18, 19, 20, 21, 22]. Colorimetric LAMP employs metal ion indicators or pH‐sensitive dyes that change color in response to DNA amplification by‐products, including pyrophosphates that deplete Mg^2+^ ions and protons [23, 24]. Although these approaches provide straightforward visual readouts, their indirect detection mechanisms render them sensitive to sample matrix properties and assay conditions, such as buffer capacity, pH, and magnesium/dNTP concentrations. In contrast, fluorescent LAMP detects DNA directly through intercalating dyes but requires external fluorophore excitation, necessitating sophisticated real‐time fluorescence systems and limiting sensitivity in complex media due to background fluorescence and scattering [25].

To address these limitations, bioluminescence‐based sensors provide a promising alternative [26]. We recently developed a bioluminescent intercalating dye (“LUMID”) that allows for direct, sequence‐independent detection of dsDNA with a ratiometric bioluminescent readout. LUMID comprises covalent conjugates of the NanoLuc luciferase with intercalating dyes that, upon binding to DNA, enable bioluminescence resonance energy transfer (BRET) between NanoLuc and the dyes. This results in a blue‐to‐green color change that is both robust and easy to detect with a digital camera [27]. Due to the limited thermostability of the sensor at LAMP reaction temperatures, LUMID can be combined with LAMP only as an endpoint readout, requiring a post‐amplification transfer of reaction products. While this approach improves assay sensitivity, the required reaction transfer step increases the risk of cross‐contamination and precludes access to kinetic information that can report on nucleic acid load and assay functioning. Consequently, a thermostable LUMID (tsLUMID) system that enables real‐time monitoring of LAMP reactions would be highly desirable for diagnostic applications.

**FIGURE 1:**
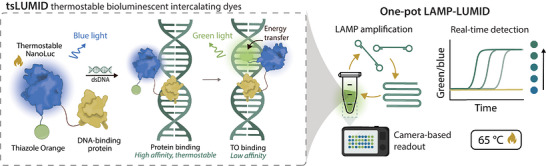
Schematic overview of the LAMP‐LUMID assay. Target DNA is rapidly amplified through LAMP at 65°C. Thermostable LUMID (tsLUMID), comprising a fusion of a nonspecific DNA‐binding protein and a thermostable NanoLuc (tsNLuc) conjugated to the intercalating dye Thiazole Orange, can bind to the LAMP amplicons, which causes a blue‐to‐green bioluminescent color change. High‐affinity and thermostable binding is mediated by the DNA‐binding protein, increasing the effective concentration of dsDNA to promote dye binding. Reactions can be monitored in real time at 65°C, using a simple camera‐based readout suitable for point‐of‐care diagnostics.

In this work, we report re‐engineered LUMID sensors that are highly thermostable and easily integrated with LAMP for one‐pot detection of nucleic acids. This tsLUMID variant comprises a thermostable NanoLuc luciferase conjugated to the intercalating dye Thiazole Orange (TO) and genetically fused to a nonspecific, thermostable DNA‐binding protein that mediates high‐affinity DNA binding, increasing the effective DNA concentration to facilitate TO binding and BRET at elevated temperatures (Figure 1). Additionally, a caged luciferin substrate with esterase‐controlled release kinetics is incorporated to allow for prolonged bioluminescent monitoring. Using these advances, tsLUMID can readily be incorporated in a LAMP reaction to monitor DNA amplification in real time through a blue‐to‐green bioluminescent color change. As a proof of concept, LAMP‐LUMID assays were designed to target the most prevalent cancer‐associated HPV subtypes 16 and 18. Using a simple camera‐based readout, we demonstrate the assay performance in clinical samples, showcasing LAMP‐LUMID as a simple and sensitive platform for rapid molecular diagnostics.

## 2 Results and Discussion

### 2.1 Thermostable Bioluminescent Signal

**FIGURE 2:**
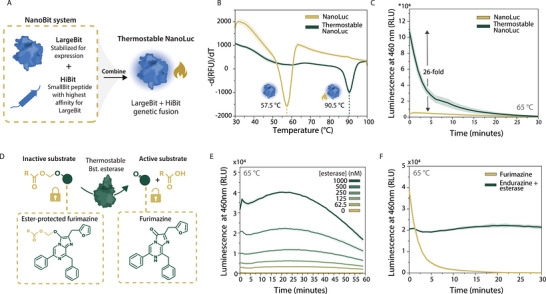
Thermostable NanoLuc and controlled substrate release enable a stable, prolonged bioluminescent readout at 65°C. (A) Development of thermostable NanoLuc using the NanoBit system. LargeBit and the highest‐affinity SmallBit (left) are genetically fused to create a more thermostable NanoLuc variant (right). (B) Thermal shift assay comparing the melt curves of NanoLuc (yellow) and thermostable NanoLuc (green). Proteins (5 µM) were combined with 2000× diluted SYPRO‐Orange dye. Fluorescence intensity was monitored at increasing temperatures ranging from 30°C to 100°C in 0.5° steps, incubating for 1 min each step. Graphs represent the negative derivative of the fluorescence with respect to the temperature. (C) Bioluminescence intensity of NanoLuc (yellow) and thermostable NanoLuc (green) at 65°C. Proteins (1 nM) were combined with 1000× diluted furimazine substrate and blue‐light (460 nm) luminescence was measured during 30 min. (D) Graphical representation of the controlled substrate release system. The inactive ester‐protected furimazine (left) is hydrolyzed by a thermostable esterase to liberate the active furimazine substrate (right). (E) Luminescence intensity using different concentrations of esterase. With 100× diluted Endurazine and 62.5 nM—1 µM of esterase, 1 nM of tsNLuc was combined, after which the luminescence intensity was monitored for 60 min at 65°C. (F) Comparison between esterase‐mediated substrate release (500 nM esterase, 100× diluted Endurazine) and the already active substrate (2000× diluted furimazine), using 1 nM of tsNLuc at 65°C. For all graphs, data represents mean ± standard deviation of *n* = 3 technical replicates.

Real‐time bioluminescent monitoring of LAMP amplification requires a thermostable luciferase that is active at reaction temperatures of 60°C–65°C. Although several hyper thermostable luciferases exist [28, 29, 30], we chose to retain the previously used NanoLuc luciferase, considering its high brightness and effective spectral overlap with the intercalating dye TO. Efforts in creating a stable split NanoLuc system have identified mutations that stabilize both parts and tune their mutual affinity [31]. Genetically fusing the highest‐affinity split pair was recently reported to strongly increase the melting temperature of the luciferase [32]. To test whether this thermostable NanoLuc (tsNLuc) also retains its enzymatic activity at elevated temperatures, we incorporated the 15 amino acid mutations found in LargeBit and the 6 amino acid mutations found in the highest affinity SmallBit into the native NanoLuc sequence (Figure 2A). This tsNLuc protein was expressed in *Escherichia coli* and purified using nickel affinity chromatography to obtain pure protein in high yields (Figure S1, 100 mg/L culture).

The melting temperature of tsNLuc was evaluated through a thermal shift assay, in which unfolding of the protein can be monitored through an increase in fluorescence caused by dye binding to exposed hydrophobic residues. To this end, we measured the fluorescence intensity of native NLuc and tsNLuc (5 µM) combined with 2000 × diluted SYPRO orange dye at increasing temperatures (35°C–100°C) and subsequently computed the negative derivative of the fluorescence with respect to the temperature to obtain melt curves (Figure 2B). In line with previous research, we observed melting temperatures of 57.5°C and 90.5°C for NLuc and tsNLuc, respectively, showing a 33°C increase in melting temperature upon introducing the stabilizing mutations. To investigate whether tsNLuc also retains its activity at LAMP reaction temperatures, we measured its bioluminescence intensity at 65°C using 1 nM of luciferase (tsNLuc and NLuc) and 1000× diluted furimazine substrate (Figure 2C). While both luciferases were similarly active at room temperature (Figure S2A), only tsNLuc produced luminescence at 65°C, confirming that the stabilizing mutations also preserve enzymatic activity at higher temperatures. Furthermore, we observed a shorter luminescence half‐life at LAMP temperatures compared to room temperature (Figure S2B), which is primarily due to fast depletion of the furimazine substrate at elevated temperatures (Figure S3). Notably, the observed half‐life appears shorter than predicted from substrate stability alone, suggesting additional temperature‐induced effects such as increased enzymatic activity, resulting in accelerated substrate depletion and/or deactivating active site modifications.

Considering that this short‐lived luminescence is not ideal for real‐time monitoring, we investigated whether we could also achieve a stable luminescent signal at higher temperatures. For live‐cell imaging, ester‐protected luciferin substrates have been developed that are activated through cleavage by cellular esterases, leading to a steady release of substrate throughout the experiment. We hypothesized that by using an ester‐protected substrate along with a thermostable esterase, substrate release can be regulated to allow for prolonged bioluminescent monitoring under LAMP reaction conditions (Figure 2D). Considering LAMP reaction temperatures, we opted for a thermostable esterase with a temperature optimum of 65°C–70°C (Bst. esterase), derived from the same thermophilic organism as the DNA polymerase used for LAMP. Since its activity towards different kinds of esters is not well characterized, two commercial ester‐protected furimazine substrates (Endurazine and Vivazine) were tested that differ in the bulkiness of their tail (R) group. We found that both substrates showed no background luminescence and were hydrolyzed at only slightly different rates to produce a similar bioluminescent output (Figure S4), reflecting the catalytic promiscuity of the esterase as well as the suitability of both substrates.

To determine the optimal conditions for substrate release during LAMP, varying concentrations of esterase enzyme were added to 1 nM tsNLuc and 100× diluted Endurazine substrate, and subsequently monitored at 65°C for 60 min (Figure 2E). We found that higher concentrations of esterase initially result in a higher absolute signal due to faster hydrolysis rates, but also in an increased rate of signal decay due to substrate depletion. Reducing the amount of esterase then results in a lower but more stable signal over time. Compared to the unprotected furimazine substrate, esterase‐mediated deprotection of Endurazine produces a much more stable luminescent signal suitable for real‐time monitoring (Figure 2F).

### 2.2 tsLUMID Sensors

**FIGURE 3:**
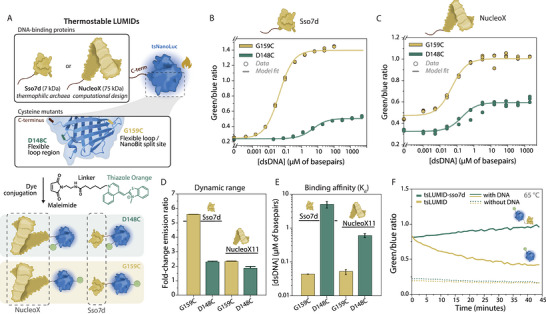
tsLUMID sensor design and characterization. (A) The thermostable LUMID sensors (tsLUMIDs) consist of thermostable NanoLuc (tsNLuc) genetically fused to an Sso7d or NucleoX11 DNA‐binding protein. Within tsNLuc's loop residues D148 and G159, single‐cysteine mutations are incorporated to allow conjugation of a maleimide‐activated Thiazole Orange dye, creating the final tsLUMID sensor proteins. (B/C) Bioluminescence titrations with dsDNA of the different DNA‐binding proteins (Sso7d, NX11) and dye positions (D148C green, G159C yellow) combinations. The sensor response was measured in the presence of salmon sperm dsDNA ranging from 400 pM—5 mM, dependent on the affinity of the tested sensor. Experiments were performed in technical replicates with *n* = 2 (Sso7d) or *n* = 3 (NX11) independent preparations of the dsDNA, with 1 nM of sensor protein and 30 min of incubation. Data was fitted using a Langmuir equation, and fitting parameters can be found in Figure S11. Individual data points are represented as circles and the model fit as solid lines. (B) Sensor response curve of Sso7d variants. (C) Sensor response curve of NucleoX11 variants. (D/E) Maximal change in emission ratio and affinity for dsDNA of all sensor variants. Bars represent average values ± standard deviation. (F) Luminescence measurements at 65°C of tsLUMID‐Sso7d (green) and tsLUMID (yellow), either with (solid) or without (dashed) DNA. tsLUMID‐Sso7d and tsLUMID (1 nM) were combined with sheared salmon sperm dsDNA (10 µM for tsLUMID‐sso7d and 7.7 mM for tsLUMID), Bst. esterase (250 nM) and Endurazine substrate (100×), and incubated for 30 min at room temperature prior to measurement. Lines represent single measurements.

LUMID's binding to dsDNA is governed by the interaction of the intercalating dye TO with DNA, which is temperature‐dependent and not stable at 65°C to allow for use during LAMP [33]. We therefore sought to strengthen and stabilize the DNA‐LUMID interaction at elevated temperatures by introducing an additional DNA‐binding protein. Considering that the geometry of the protein‐DNA complexes relative to the intercalating dye might affect the efficiency of dye binding and BRET, we chose to screen different proteins in combination with different dye positions (Figure 3A). Two highly thermostable (*T*_m_ > 90°C) DNA‐binding proteins were tested, Sso7d and NucleoX11, that differ in size and DNA‐binding geometry. Sso7d is a small, 7 kDa protein derived from thermophilic archaea that binds nonspecifically across the minor grove with µM‐affinity [34]. Fusions with Sso7d are known to improve the processivity of DNA polymerases by stabilizing the polymerase‐DNA interaction during PCR amplification [35]. NucleoX11 (NX11) is a 64 kDa computationally redesigned mimic of the Transcription Activator‐Like Effector (TALE) protein. It consists of multiple repeating units that electrostatically interact with the DNA major groove, and includes the native TALE N‐terminal region as a capping region to block potential cooperative assembly [36]. Because NX11 consists of simple repeats, it can be readily extended or shortened to tune binding strength or adjust fusion orientation as needed. NX11 binding strength was estimated using an electrophoretic mobility shift assay (EMSA), suggesting stronger binding than Sso7d with sub‐µM affinity (Figure S5).

To identify suitable intercalating dye positions, a pre‐screening was performed by conjugating TO to different positions within the native NanoLuc enzyme and testing the BRET response to increasing concentrations of dsDNA (Figures S6, S7 and earlier work [27]). We selected for sufficient luciferase activity, stability towards aggregation, efficient DNA binding and BRET as well as for different relative orientations with respect to the DNA‐binding protein. Based on this screening, single‐cysteine mutations were incorporated in two of tsNLuc's flexible loop regions (D148C and G159C, Figure 3A).

Sso7d and NX11 were genetically fused to the C‐terminus of the tsNLuc cysteine mutants through a short 4‐repeat GGS linker, keeping tsNLuc close to the DNA for efficient dye intercalation. The fusion proteins contained both an N‐terminal streptavidin‐tag and a C‐terminal hexahistidine‐tag to facilitate purification after expression in *E. coli*, which yielded pure proteins in sufficient yields (∼ 10 mg/L culture, Figures S8 and S9). To create the thermostable LUMID sensors (tsLUMIDs), the proteins were conjugated to a maleimide‐TO that reacts with the introduced cysteine residues to form a stable thioether bond. The maleimide derivative was pre‐synthesized from a TO‐NHS ester and an amine‐maleimide crosslinker as described previously [27]. Q‐ToF LC‐MS analysis confirmed dye labeling of the cysteine residues and showed no remaining unconjugated protein. Only for the G159C‐NucleoX11 variant, the presence peak with an apparent molecular weight close to that of the unconjugated protein suggests partial labeling (Figures S10 and S11).

The sensor response of tsLUMID‐Sso7d and ‐NX11 to dsDNA was evaluated through bioluminescence titrations with dsDNA. Increasing concentrations of sheared salmon sperm dsDNA were added to 1 nM of sensor and incubated for 30 min at room temperature, followed by the addition of furimazine substrate. For the Sso7d constructs, the D148C variant displayed an increase in green/blue ratio in the low‐µM range (*K_d,app_* = 5.1 ± 1.4 µM), while for G159C the ratio already increased in the nM‐range (*K_d,app_* = 43 ± 4.8 nM) (Figure 3B). Since the apparent *K_d_* of the G159C variant is well below reported binding affinities of Sso7d and TO (17 and ∼ 10 µM of base pairs, respectively) [34, 37], the results suggest an avidity effect in which positioning the dye at the G159C position enables bivalent, enhanced binding of Sso7d and TO to the DNA. Furthermore, the increase in green/blue ratio was larger for G159C (6‐fold) than for D148C (2‐fold). Since BRET is strongly distance dependent, this difference in emission ratio can be attributed to the distance between the dye and the active site of tsNLuc in both mutants, that is, positioning of the dye in the D148C loop leads to a larger distance to the active site compared to the G159C loop, and therefore a reduced BRET efficiency (Figure S12).

Consistent with the results of Sso7d, the G159C variant of NX11 exhibited a higher apparent affinity for dsDNA and a greater increase in green/blue ratio (*K_d,app_*_=_ 54 ± 15 nM, 2.3‐fold) compared to the D148C variant (*K_d,app_*_=_ 594 ± 11 nM, 1.8‐fold) (Figure 3C). Interestingly, the increase in green/blue ratio was smaller for G159C‐NX11 than for G159C‐Sso7d. This difference may be explained by the large size of NX11, which likely changes the relative orientation of tsNLuc and TO, leading to an increased donor‐acceptor distance and/or less favorable dipole‐dipole orientation and consequently a decreased BRET efficiency. Additionally, incomplete dye labeling of this variant cannot be fully excluded and would increase the blue background, thereby reducing the dynamic range.

Next, we assessed if the best‐performing tsLUMID variant (G159C‐Sso7d), having the largest change in emission ratio and sufficient affinity for dsDNA (Figure 3D,E), is able to maintain DNA binding at LAMP reaction temperatures. Sheared salmon sperm dsDNA was added to 1 nM of sensor protein at a concentration sufficient to just saturate the sensor proteins (10 µM and 7.7 mM in the presence and absence of Sso7d, respectively) to allow observation of temperature‐dependent decreases in affinity for both constructs. During 45 min incubation at 65°C, the tsLUMID sensor showed a slightly increasing green/blue ratio, indicating that prolonged incubation at elevated temperature does not adversely affect the DNA‐binding affinity of the sensor (Figure 3F). Without the Sso7d protein, we observed a decrease in green/blue ratio that confirms the necessity of Sso7d in maintaining DNA binding at LAMP reaction temperatures.

### 2.3 Real‐Time LAMP‐LUMID

**FIGURE 4:**
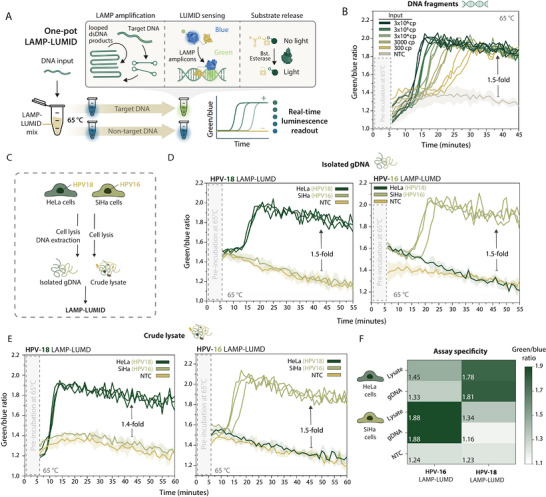
Real‐time LAMP‐LUMID for HPV detection. (A) Schematic overview of one‐pot LAMP‐LUMID assay. Target DNA is sequence‐specifically amplified through LAMP and detected by LUMID through a blue‐to‐green color change. During the reaction, substrate release is controlled through esterase‐mediated substrate release to obtain a stable signal. (B) LAMP‐LUMID assay targeting the HPV18 E7 gene using synthetic DNA fragments. (C) The performance of LAMP‐LUMID in complex sample matrices is tested through isolated genomic DNA (gDNA) and crude cell lysate from HeLa (HPV18) and SiHa (HPV16) cell lines. (D, E) LAMP‐LUMID assay targeting the HPV18 and ‐16 E7 genes using gDNA (D) and crude cell lysate (E) of HeLa (dark green) and SiHa (light green) cell lines. For all LAMP‐LUMID assays, LAMP reactions were performed at 65°C for 45–60 min with the addition of 10 nM tsLUMID, 1 µM esterase, and 100× diluted Endurazine substrate. Reactions were incubated for 5 min at 65°C prior to readout. Lines of positive reactions represent *n* = 3 technical replicates. Lines of negative reactions represent mean ± standard deviation with *n* = 3 technical replicates. Indicated fold‐changes represent fold‐change increase in green/blue ratio relative to NTC at *t* = 45 min. (F) Heatmap displaying the specificity of the LAMP‐LUMID assay for HPV18 and ‐16 detection in HeLa cells and SiHa cells. Colors represent green/blue ratio at *t* = 45 min.

Next, we explored whether tsLUMID could be integrated with LAMP to allow for real‐time amplification and bioluminescent detection of target DNA (Figure 4A). As a clinically relevant target, we chose to target high‐risk HPV subtypes, given that persistent high‐risk HPV infection is the primary cause of cervical cancer. In settings with limited access to vaccination and advanced laboratory setups, such as in many low‐income countries, there is an established need for a simple but accurate point‐of‐care test to help augment HPV screening [38]. Generally, the high‐risk subtypes HPV16 and HPV18 account for the majority (approximately 70%) of precancerous lesions, and therefore LAMP primer sets were chosen to target these subtypes [39]. Primer sequences were selected from previous research, targeting the E7 oncogene that exhibits large genetic differences with low‐risk HPV subtypes that are not associated with cancer, hence minimizing the risk of cross‐reactivity between subtypes [40, 41]. Importantly, selected primer sets have been validated in multiple studies using non‐specific readouts to ensure specificity of amplification [42, 43, 44].

Amplification of a synthetic HPV18 E7 gene was monitored by measuring real‐time luminescence, performing LAMP reactions in buffered solution according to manufacturer's protocol, but with the addition of 10 nM of tsLUMID, 1 µM esterase and 100× diluted Endurazine substrate. During incubation at 65°C, input DNA concentrations down to 300 copies per reaction (20 aM in reaction) could be distinguished from the non‐template control (NTC) with a 1.5‐fold change in green/blue ratio within 30 min (Figure 4B). Higher target input concentrations were consistently detected earlier, demonstrating that LAMP‐LUMID detection time reflects nucleic acid load. Over a defined concentration range, a log‐linear relationship was observed (Figure S14) between the target copy numbers and the LAMP‐LUMID detection time, indicating that estimating target loads is feasible under well‐controlled conditions by using a real‐time readout. The change in emission ratio observed for the one‐pot LAMP‐LUMID was somewhat attenuated due to increased green background emission, which can be ascribed to background binding to primers and LAMP reaction conditions (Figure S15). Nevertheless, the remaining 1.5‐fold change in emission ratio is robust and sufficient for camera‐based detection. For comparison, the HPV18 LAMP assay was also performed using the intercalating dye SYBR green I as a fluorescent readout, which resulted in a comparable sensitivity and detection time with a slightly higher dynamic range (∼ 4‐fold, Figure S16), but lacked the internal signal calibration of a ratiometric readout and required more sophisticated instrumentation. Following these results, we also set up a HPV16 assay using synthetic E7 genes, which showed a comparable performance (Figure S17). For both assays, additional measurements with decreasing amounts of input DNA were performed to estimate the limit of detection, which was found to be around 150 and 75 copies per reaction for HPV16 and HPV18, respectively (Figure S18).

To assess the performance of LAMP‐LUMID in more complex samples, we tested our assay using genomic DNA (gDNA) extracted from cells containing integrated copies of either HPV18 (HeLa cells) or HPV16 (SiHa cells) genome (Figure 4C). Using similar conditions as with the synthetic genes, LAMP‐LUMID unambiguously identified HPV16 and HPV18 only in SiHa and HeLa cells, respectively (Figure 4D,F). This demonstrates that, despite the nonspecific readout, our assay allows for discrimination of genetic differences between closely related viral subtypes. Similar results were obtained by using crude cell lysate (Figure 4E,F), showing that LAMP‐LUMID performs equally well without the need for laborious DNA extraction. Although target input concentration were likely high based on the fast detection times (10–15 min), no apparent increase in background due to the presence of genomic DNA was observed. The increase in emission ratio was very similar to that observed for synthetic targets, showcasing the robustness of LAMP‐LUMID as a nucleic acid diagnostic platform.

Since one‐pot integration of sensing components can interfere with LAMP amplification and thereby reduce assay sensitivity, as seen with intercalating dyes and CRISPR‐based readouts [45, 46], we benchmarked the one‐pot LAMP‐LUMID assay against our previously reported two‐step assay targeting SARS‐CoV‐2 by using identical LAMP primer sets [27]. The one‐pot LAMP‐LUMID assay detected target input concentrations down to 30 copies per reaction (2 aM in reaction), whereas the two‐step LAMP‐LUMID assays detected down to 3000 copies per reaction (200 aM in reaction) with stochastic responses at lower input concentrations (Figure S19). These findings indicate that one‐pot integration of LUMID does not compromise assay sensitivity. The apparent increase in detection limit can be attributed to differences in reaction conditions, such as inclusion of an additional heating step to promote primer annealing and template denaturation.

### 2.4 Clinical Validation LAMP‐LUMID With Camera‐Based Readout

**FIGURE 5:**
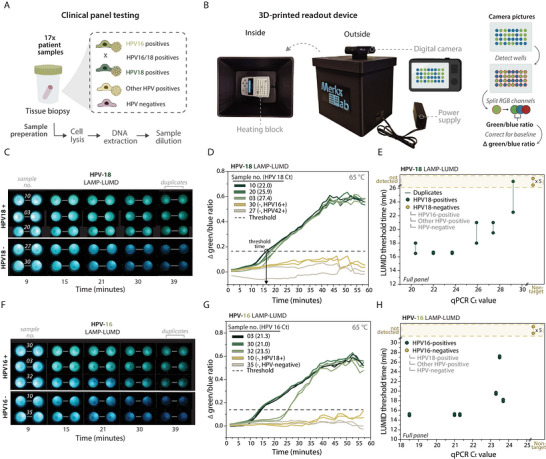
Clinical validation of LAMP‐LUMD with camera‐based readout. (A) Schematic overview of the clinical sample panel and sample preparation steps. (B) Experimental setup as used for the camera‐based readout of the LAMP‐LUMID assay. Setup consists of a dark 3D‐printed enclosure with a small heating block inside and a digital camera on top. Schematic (right) shows the camera picture processing workflow. (C, F) Visual representation of a subset of patient samples for HPV18 (C) and HPV16 (F), showing the blue/green intensity at several points in time. Two consecutive wells, connected with a dash, represent technical duplicates. (D, G) Δ green/blue traces over time for HPV18 (D) and HPV16 (G) extracted from camera pictures shown in panels (C) and (F). Camera pictures were taken continuously with an exposure time of 90 s. Traces of other samples are shown in Figure S21. Dashed line indicates threshold based on mean Δ green/blue and the variance among all reactions (see methods). LUMID threshold time corresponds to the timepoint at which the Δ green/blue ratio first surpasses the set threshold. Lines represent technical replicates with *n* = 2. (E/H) Overview of LAMP‐LUMID outcomes for all samples tested for HPV18 (E) and HPV16 (H). Data compare LUMID threshold time to the qPCR cycle threshold value (*C*_t_) as an indicator for target load. Circles represent individual data points, and duplicates are connected with vertical lines. All reactions were performed with 1 µL of 10× diluted patient sample as input, using standard LAMP conditions with the addition of 10 nM tsLUMID, 2 µM esterase, and 100× diluted Endurazine in a single 25 µL reaction.

To further validate the LAMP‐LUMID platform, we evaluated its performance on a small clinical panel (*n* = 17) of HPV‐positive and ‐negative DNA isolates from tissue biopsies, obtained as part of routine HPV diagnostics (Figure 5A). The panel included HPV16‐positives, HPV18‐positives, HPV16/18 double‐positives and various other high‐ and low‐risk HPV subtypes, with varying target loads determined by a parallel qPCR assay. To allow for straightforward signal recording in a low‐resource setting, a heating block in a 3D‐printed dark enclosure was used in combination with a standard digital camera (Figure 5B). The setup was first tested using the same conditions as in Figure 4D, but using the camera pictures as readout. Green‐over‐blue ratios were obtained by automatically detecting the wells and calculating the mean intensity of green and blue channels in each well. We observed an overall decrease in green/blue ratio that coincides with the decrease in signal intensity, which suggests that the image sensor is more prone to saturation by blue light compared to green light. By using a water control to correct for this background decrease (Δ green/blue ratio), we obtained similar response curves compared to the plate reader assays (Figure S20).

Using the camera‐based setup, we tested the full clinical panel using 1 µL of DNA isolate in a 25 µL LAMP‐LUMID reaction. Please note that due to the high DNA content in tissue samples, DNA isolates were diluted 10 times prior to measurement to avoid direct, nonspecific detection of cellular DNA. Samples were considered positive if the Δ green/blue ratio surpassed a self‐set threshold value determined by the mean Δ green/blue ratio plus 8 times the standard deviation of all samples at *t* = 6 min. The reported LUMID threshold time corresponds to the timepoint at which the Δ green/blue ratio first surpasses the set threshold value (Figure 5D). The HPV18 LAMP‐LUMID assay correctly identified all HPV18‐positive samples (*n* = 6) in under 30 min (Figure 5C–E). Only for the sample with the lowest target load, a stochastic assay response was observed, with one out of two replicates showing an increase in signal above the threshold (Figure 5E). All HPV16‐positive samples (*n* = 6) were also found positive by the HPV16 LAMP‐LUMID assay in under 30 min, with 10/12 duplicates already surpassing the threshold within 20 min (Figure 5F–H). Although a trend towards longer detection times for samples with higher qPCR cycle threshold (*C*_t_) values was observed, extracting quantitative information is precluded due to inter‐sample variability inherent to clinical material. Several samples were tested in duplex with both assays (Figure 5C,D,F,G samples 10, 03, and 30) to confirm orthogonal assay response, that is, the HPV18‐ and HPV16‐positive samples were only detected by their corresponding assay whilst the double‐positive is detected by both. Importantly, all HPV18‐negative and HPV16‐negative samples (*n* = 5 each) were correctly identified as negative, indicating minimal non‐specific binding to cellular DNA and no cross‐reactivity with other HPV subtypes. This specificity is particularly important for HPV diagnostics, considering the different clinical outcome of infection with low‐ or high‐risk HPV. Although the detection limit of our assay seems slightly higher than the parallel qPCR assays, this may be due to lower DNA input as a result of sample pre‐dilution and lower input volumes, rather than the sensitivity of our method. These results showcase that LAMP‐LUMID enables fast diagnosis of viral subtypes with high sensitivity and specificity, using simple equipment suitable for a point‐of‐need diagnostic setting.

## 3 Conclusion

In this study, we have developed tsLUMID sensors that integrate with LAMP amplification as a rapid and simple platform for molecular diagnostics. Introducing a thermostable NanoLuc luciferase and a thermostable DNA‐binding protein enabled both luciferase activity and DNA binding at LAMP reaction temperatures (60°C–65°C). Systematic screening of different DNA‐binding proteins and intercalating dye positions yielded a sensor variant with a sufficient affinity for dsDNA (*K_d,app_* 43 nM of basepairs) and a large dynamic range (6‐fold increase in green/blue ratio). To allow for real‐time monitoring, we also incorporated a caged luciferin substrate that is slowly released, generating a prolonged bioluminescent signal at elevated temperatures. The optimized LUMID sensors and substrate were successfully integrated with LAMP into a one‐pot assay for detection of cancer‐associated HPV subtypes with attomolar sensitivity within ∼ 30 min. Finally, we showed that the LAMP‐LUMID assay enables robust detection of HPV subtypes in clinical samples using a simple camera‐based readout, demonstrating its application for infectious disease diagnostics in a resource‐limiting setting.

LAMP‐LUMID's performance is comparable to that of established molecular diagnostic assays. We demonstrated reliable detection of HPV16 and ‐18 target DNA down to ∼ 150 and 75 copies per reaction, respectively, comparable to other reported point‐of‐care HPV assays [47] and approaching the sensitivity of the benchmark qPCR‐based assay (50 copies per reaction) [48], but using cheap and simple equipment. Unlike other integrated molecular diagnostic tests, one‐pot LAMP‐LUMID did not show reduced test sensitivity compared to the corresponding two‐step assay [27]. Sensitivity in clinical samples could likely be further improved by increasing the sample input volume, using sample matrices with a lower background of cellular DNA, such as swabs or urine: samples types that are generally preferred as they are considerably less invasive than tissue biopsies. The typical time‐to‐result was under 30 min, which is significantly faster than conventional qPCR workflows and comparable to other point‐of‐care tests [15, 16, 21, 24]. Because the assay relies on the specificity of the nucleic acid amplification reaction, LUMID functions as an off‐the‐shelf probe that can be readily incorporated without the need for target‐specific modifications. The ability to monitor the LAMP reaction in real‐time in a one‐pot assay with tsLUMID avoids the risk of cross‐contamination, which is typically a problem with common lateral flow assays or two‐step CRISPR‐based assays [49]. To expand LAMP‐LUMID towards RNA targets, a thermostable reverse transcriptase could be included to enable direct, one‐pot direction of RNA without additional workflow steps.

Enhancing the thermostability of the LUMID sensor was essential for integration with LAMP, but offers broader advantages. The tsLUMID sensors can be modularly integrated with other isothermal amplification techniques that operate at elevated temperatures, for example, helicase‐dependent amplification or strand‐displacement amplification, provided they do not produce spurious DNA amplification [50]. We further hypothesize that the current LAMP‐LUMID assay could enable integrated thermal lysis of viruses or bacteria, potentially eliminating the need for separate sample preparation steps. The high stability of the assay components allows for easy production and storage, and might also facilitate lyophilization for increased shelf life. Finally, the controlled substrate‐release system could prove valuable in applications that are currently limited by luciferin substrate depletion, such as in continuous monitoring.

Application in low‐resource settings requires assays to be robust, cost‐effective and integrated in a user‐friendly device, along with the development of a dedicated reader. The simplified one‐pot workflow facilitates device integration and as most LUMID components are used at low concentrations, their contribution to overall costs is minimal, rendering the assay competitive with existing LAMP‐based assays with the added advantage of low‐cost readout equipment (Supporting Note). Although LAMP‐LUMID requires a more complex reaction mixture compared to conventional LAMP approaches, the real‐time ratiometric readout ensures robustness against variability between individual reaction components. The ratiometric signal calibrates for variations in absolute intensity that may arise from differences in enzyme activity or substrate stability, while the real‐time analysis avoids dependence on a fixed reaction time or threshold ratio that may be affected by changes in LAMP efficiency or DNA binding. Collectively, these features position LAMP‐LUMID as a robust, sensitive, and accessible nucleic acid detection platform that is particularly attractive for point‐of‐care diagnostics.

## Author Contributions

**Yosta de Stigter**: conceptualization, writing – original draft, investigation, writing – review and editing, supervision, data curation, methodology. **Harmen J. van der Veer**: writing – review and editing, supervision, investigation. **Sterre de Lignie**: investigation, writing – review and editing. **Robbert J. de Haas**: investigation, writing – review and editing. **Renko de Vries**: supervision, writing – review and editing. **Joost P. H. Schoeber**: supervision, writing – review and editing, funding acquisition. **Anne J. M. Loonen**: supervision, resources, writing – review and editing, funding acquisition. **Adriaan J. C. van den Brule**: resources, writing – review and editing. **Maarten Merkx**: conceptualization, funding acquisition, writing – review and editing, supervision.

## Conflicts of Interest

The authors declare no conflicts of interest.

## Supporting information




**Supporting File**: anie72751‐sup‐0001‐SuppMat.pdf.

## Data Availability

The data that support the findings of this study are available from the corresponding author upon reasonable request.
