# Supplementary material for: Thermostable Bioluminescent Intercalating Dyes for Real‐Time, Integrated Nucleic Acid Amplification and Detection
Source: Angew Chem Int Ed Engl. 2026 May 21;65(29):e6796450. doi: 10.1002/anie.6796450 (PMC13360676; doi:10.1002/anie.6796450)
Supplement: Supplementary file 1 — Supporting File: anie72751‐sup‐0001‐SuppMat.pdf. [file ANIE-65-e6796450-s001.pdf]

Supplementary information

Thermostable Bioluminescent Intercalating Dyes For Real-Time, Integrated Nucleic Acid Amplification And Detection

Yosta de Stigter[a,b,d], Harmen J. van der Veer[a,b], Sterre de Lignie[a,b], Robbert J. de Haas[c], Renko de Vries[c], Joost P.H. Schoeber[d], Anne J.M. Loonen[d,e], Adriaan J.C. van den Brule[e] and Maarten Merkx*[a,b]

[a] Yosta de Stigter, Harmen J. van der Veer, Sterre de Lignie and Maarten Merkx
Laboratory of Chemical Biology, Department of Biomedical Engineering, Eindhoven University of Technology
Eindhoven, 5612 AZ, The Netherlands
m.merkx@tue.nl

[b] Yosta de Stigter, Harmen J. van der Veer, Sterre de Lignie and Maarten Merkx
Institute for Complex Molecular Systems, Eindhoven University of Technology
Eindhoven, 5612 AZ, The Netherlands

[c] Robbert J. de Haas and Renko de Vries
Department of Physical Chemistry and Soft Matter, Wageningen University and Research
Wageningen, 6708 WE, The Netherlands

[d] Yosta de Stigter, Joost P.H. Schoeber and Anne J.M. Loonen
Research Group Applied Natural Sciences, Fontys University of Applied Sciences
Eindhoven, 5612 AP, The Netherlands

[e] Anne J.M. Loonen and Adriaan J.C. van den Brule
Pathologie-DNA, Lab for Molecular Diagnostics, Location Jeroen Bosch Hospital
's-Hertogenbosch, 5223 GZ, The Netherlands.

Table of Contents

Methods 4

Supplementary note production costs LAMP-LUMID 7

Supplementary figures 9

Supplementary Figure S1. SDS-PAGE analysis of expression and purification thermostable NanoLuc. 9

Supplementary Figure S2. Comparison luminescence intensity of NanoLuc, thermostable NanoLuc and thermostable NanoLuc at 60 °C 9

Supplementary Figure S3. Stability of Furimazine substrate at LAMP reaction temperature 10

Supplementary Figure S4. Comparison of esterase-mediated deprotection of Vivazine and Endurazine substrates 10

Supplementary Figure S5. Electrophoretic mobility shift assay to compare NX11 and Sso7d DNA binding. 11

Supplementary Figure S6. Pre-screening of dye positions within the NanoLuc luciferase 11

Supplementary Figure S7. Effect of cysteine mutations on NanoLuc luciferase activity 12

Supplementary Figure S8. Expression and purification of tsNLuc-Sso7d proteins 13

Supplementary Figure S9. Expression and purification of tsNLuc-NX11 proteins 14

Supplementary Figure S10. ESI-QToF mass spectra of tsNanoLuc-Sso7d proteins 15

Supplementary Figure S11. ESI-QToF mass spectra of tsNanoLuc-NX11 proteins 15

Supplementary Figure S12. Schematic overview of NanoLuc structure 16

Supplementary Figure S13. Model fits of different tsLUMID variants 17

Supplementary Figure S14. Log-linear correlation between input target concentrations and LAMP-LUMID detection time 18

Supplementary Figure S15. Background controls LAMP-LUMID 18

Supplementary Figure S16. Intercalating dye-based real-time LAMP targeting HPV18 E7 19

Supplementary Figure S17. LAMP-LUMID assay targeting the HPV16 E7 gene using synthetic DNA fragments 19

Supplementary Figure S18. Real-time LAMP-LUMID targeting HPV16 and HPV18 E7 for LOD determination 20

Supplementary Figure S19. Real-time LAMP-LUMID targeting Sars-CoV-2 N-gene 20

Supplementary Figure S20. Camera-based LAMP-LUMID on quality control material 21

Supplementary Figure S21. Green/blue traces over time for HPV16 and HPV18 extracted from camera pictures 21

Supplementary Figure S22. DNA and amino acid sequence of tsNanoLuc 22

Supplementary Figure S23. DNA and amino acid sequence of thermostable NanoLuc and Sso7d fusion proteins 22

Supplementary Figure S24. DNA and amino acid sequence of thermostable NanoLuc and NucleoX11 fusion proteins 24

Supplementary Figure S25. MatLab script to extract green-over-blue ratios from camera pictures 25

Supplementary tables **26**

Supplementary Table S1. Mutagenesis primers 26

Supplementary Table S2. HPV16 and HPV18 E7 synthetic genes 26

Supplementary Table S3. LAMP primer sets targeting HPV16 and HPV18 E7 27

Supplementary Table S4. Overview clinical samples 28

Methods

Cloning. A synthetic gene encoding thermostable NanoLuc (tsNLuc) with a C-terminal hexahistidine-tag was ordered from Integrated DNA Technologies (IDT) and inserted into a linearized pET28a expression vector through Gibson Assembly, using the HiFi DNA assembly kit (New England Biolabs) according to manufacturer's instructions. To create the thermostable NanoLuc cysteine mutants, site-directed mutagenesis to introduce new cysteine residues was carried out using the QuikChange Lightning Site-Directed Mutagenesis kit (Agilent), using specific primers according to the manufacturer's instructions (supplementary Table S1). For the NX11 variants, gene fragments encoding strep-tag-NanoLuc-Native N-Terminus-NX1 with a C-terminal BsaI cleavage site and NX5 flanked with two BsaI cleavage sites were ordered from IDT. Mutagenesis PCR on the NX5 BsaI cleavage sites was performed using specifically designed primers, creating a new NX5 insert with an unique combination of BsaI cleavage sites to facilitate sequential assembly of two blocks into 11 NX repeats. A golden-gate compatible pET24(+) vector containing BsaI cleavage sites and a C-terminal hexahistidine-tag was gifted by Rob de Haas (Wageningen University and Research). Restriction and ligation of the inserts into the plasmid was performed via the Golden-gate assembly method according to manufacturer's instructions (New England Biolabs). Using overlap extension PCR, the pET24(+) vector with NX11 was linearized and the genes for tsNLuc cysteine mutants were isolated. Subsequent assembly using Gibson Assembly yielded pET24(+) vectors containing tsNLuc-NX11. For sso7d, a synthetic gene was ordered (IDT) and directly inserted into linearized pET24(+) vectors, already containing the tsNLuc cysteine mutants. All cloning results were confirmed by Sanger sequencing (Azenta Life Sciences). An overview of the DNA and protein sequences can be found in supplementary Figures S22-24.

Protein expression and purification. The plasmids encoding tsNLuc and tsNLuc-NX11/Sso7d were transformed into chemically competent *E. coli* BL21 (DE3) and cultured in 2YT medium (16 g peptone, 5 g NaCl, 10 g yeast extract per liter) supplemented with 50 µg/mL kanamycin. At OD_{600} = 0.6, protein expression was induced using 0.5 mM (tsNLuc-NX11/Sso7d) or 1 mM (tsNLuc) isopropyl β-D-1-thiogalactopyramoside (IPTG) overnight at 20°C. Subsequently, cells were harvested by centrifugation (10 min, 4000xg, 4°C). For tsNLuc, cells were lysed using Bugbuster protein extraction reagent (Novagen), supplemented with Benzonase endonuclease (Novagen). For tsNLuc-NX11/Sso7d, cells were resuspended in 10 mL pre-chilled lysis buffer (500 mM NaCl, 1 mM TCEP, 50 mM Tris pH 8.0) per gram cell pellet, supplemented with Benzonase endonuclease (Novagen). The cells were lysed through 15 minutes of sonication on ice, using a 15 sec duty cycle at 50% amplitude (Qsonica Q500) and subsequently centrifuged at 40,000xg for 30 min at 4°C. For purification, the supernatant was run twice over a Ni^{2+}-NTA affinity column, followed by three washing steps consisting of 1 column volume (CV, 10 mL) of basic buffer (1xPBS, 370 mM NaCl, 20 mM imidazole, 10% (v/v) glycerol, pH 7.4), 3 CV of high salt buffer (1xPBS, 2 M NaCl, 20 mM imidazole, 10% (v/v) glycerol, pH 7.4) and 0.5 CV of basic buffer. Proteins were eluted using 1 CV of elution buffer (1xbasic buffer, 230 mM imidazole). The resulting elution fraction was purified using Strep-Tactin XT (Iba) purification, according to manufacturer's instructructions. SDS-PAGE was used to determine protein purity. Correct protein mass was confirmed by Q-ToF LC-MS (WatersMassLynx v4.1), using MagTran v1.03 for MS deconvolution. Mass spectra were obtained using a 1 µL injection volume, containing 0.1 mg/mL of protein in Q-ToF buffer (MilliQ, 0.1% formic acid). Purified proteins were stored in strep elution buffer at -80 °C until conjugation.

Conjugation of maleimide-activated dyes to tsNLuc-NX11/Sso7d. For conjugation of the maleimide-activated TO, the proteins were reduced through incubation with 5 mM of TCEP for 1 hour at room temperature in with continuous shaking at 500 rpm and subsequently buffer exchanged to a sodium phosphate buffer (100 mM $NaPO_4$, 25 µM TCEP, pH 7.0) using a PD-10 desalting column (GE Healthcare). Then, the maleimide-activated TO was added in a 10-fold molar excess to 10 µM of reduced NanoLuc and allowed to react for 2 hours at room temperature with continuous shaking at 500 rpm. Maleimide-activated TO was prepared as described previously[27]1. The NanoLuc-dye conjugates were purified by a PD-10 desalting column to remove excess dye and simultaneously buffer exchanged to PBS (100 mM NaPi, 150 mM NaCl, pH 7.2). The coupling efficiency and correct mass of the NanoLuc-dye conjugates were confirmed by Q-ToF LC-MS (WatersMassLynx v4.1), using MagTran v1.03 for MS deconvolution. Mass spectra were obtained using a 2 µL injection volume, containing 0.1 mg/mL of protein in Q-ToF buffer (MilliQ, 0.1% formic acid, 10% acetonitrile).

Thermal shift assay. Thermal shift assays were performed using 5 µM of protein and 500x diluted SYPRO orange dye (Invitrogen) in a total volume of 25 µL PBS buffer (pH 7.4) in a white skirted 96-well plate (VWR). Fluorescence ($\lambda_{excitation}$ = 450-490 nm, $\lambda_{detection}$ = 560-580 nm) was monitored every 0.5 °C in the range of 25 to

100 °C using the Bio-Rad CFX384 Real-Time qPCR system. Proteins were incubated for 20 seconds at each temperature prior to measurement. Melt curves were obtained through the Bio-Rad CFX384 software, by computing the negative derivative of the fluorescence with respect to the temperature.

Kinetic luminescent assays at 65 °C. Kinetic luminescent assays were performed using 1 nM of luciferase and 1000-2000x diluted furimazine substrate (Promega, N1110) in a total volume of 20 µL PBS buffer (pH 7.4, 0.1% (w/v) BSA) in a VWR white 96-well skirted PCR plate. The assay components were first heated to 65 °C, and then immediately placed in a plate reader (FluoStar Omega, Isogen life Science), pre-warmed at 65 °C, to measure luminescence intensity at 460 nm. Kinetic luminescent assay with dsDNA were performed using similar conditions, but with the addition of 10 µM (tsNLuc-Sso7d) and 7.7 mM (tsNLuc) of sheared salmon sperm dsDNA (Thermo Fisher). Luminescence intensity was measured at 460 nm and at 520 nm (3 mm spiral, gain 3000), and the green/blue ratio was calculated by dividing bioluminescent emission at 520 nm by emission at 460 nm.

Controlled substrate release. Bst esterase was obtained from Merck Life Science (cat. # 79302), diluted in storage buffer (30 mM Tris-base, 85 mM NaCl, 50% (v/v) glycerol, pH 7.4) and stored at -30 °C. The ester-protected substrates Endurazine and Vivazine were ordered from Promega (N2590). Assays were performed using similar conditions as with the kinetic luminescent assays, with the addition of 61 nM – 1 µM of Bst esterase and 100x diluted Endurazine or Vivazine instead of furimazine.

Bioluminescent titrations with dsDNA. Titrations with dsDNA were performed at sensor protein concentrations of 1 nM in a total volume of 20 µL PBS buffer (pH 7.4, 0.1% (w/v) BSA, 5% DMSO) in a PerkinElmer flat white 384-well optiplate. Sheared Salmon Sperm dsDNA fragments of ~ 2000 bp were ordered from Thermo Fisher and diluted to a concentration range of 400 pM – 5 mM, measured in terms of the number of base pairs. After incubation of sensor proteins with dsDNA fragments for 30 minutes at room temperature, Furimazine substrate (Promega, N1110) was added at a final dilution of 1:1000. Luminescence spectra were recorded in a plate reader (Tecan Spark 10M) between 398 nm and 653 nm with a step size of 15 nm, a bandwidth of 25 nm and an integration time of 100 ms. The green/blue ratio was calculated by dividing bioluminescent emission at 533 nm by emission at 458 nm. Sensor response curves were fitted in Origin (2020) using a 1:1 Langmuir binding model with offset to determine an apparent affinity, using the equation below. The fitting parameters of all titration curves can be found in supplementary Figure S13.

$$\frac{green}{blue}\,ratio = \frac{green}{blue}ratio\_start + (\frac{green}{blue}ratio\_end - \frac{green}{blue}ratio\_start) * \frac{[dsDNA]}{k + [dsDNA]}$$

$k = Michaelis\ constant$

Real-time LAMP-LUMID. Sequences for LAMP primers targeting the HPV16 and HPV18 E7 gene were retrieved from Saetiew et al.[41] and ordered PAGE-purified from Integrated DNA Technologies (IDT)[2]. Primer and target sequences can be found in supplementary Table S3. For LAMP reactions, primers were combined in a 10 x concentrated stock, containing 16 µM of inner primers, 2 µM of outer primers and 8 µM of loop primers. Synthetic HPV18 and HPV16 E7 genes were PCR amplified from HeLa and SiHa cell lines, respectively. The amount of E7 gene fragments was determined by absorbance measurements at 260 nm, after which stocks were serially diluted to 25 x concentrated stocks ranging from 500 aM – 5 pM. Positive LAMP reactions were assembled by combining 1.25 x isothermal amplification buffer (NEB), 7.5 mM of $MgSO_4$ (NEB), 1.75 mM of dNTPs (NEB), 1.25 x LAMP primer mix and 1.25 x target DNA in a total volume of 20 µL, and heating the mixture for 3 min at 95 °C. For the non-template control, similar conditions were used, only interchanging the target DNA for MilliQ water. Reactions were kept on ice during the full assembly process. To initiate the LAMP reactions, 5 µL of a 5x enzyme mix containing Bst 2.0 polymerase (1.6 U/µL, NEB), tsLUMID (50 nM), Bst esterase (5 µM) and Endurazine substrate (20x diluted) was added to the reaction mixture, mixed through pipetting and sealed using a drop of transparent mineral oil. The reactions were first heated to 65 °C for 5 min, and then immediately placed in a plate reader (FluoStar Omega, Isogen life Science), pre-warmed at 65 °C, to measure luminescence intensity at 460 nm and 520 nm (3 mm spiral, gain 3000), every minute during 45-60 minutes. The green/blue ratio was calculated by dividing bioluminescent emission at 520 nm by emission at 460 nm.

LAMP-LUMID with cell isolates. Genomic DNA of SiHa and HeLa cells was extracted from 10^5 cells in a final volume of 100 µL, using the SwiftX™ Hi-Sense kit (Xpedite Diagnostics) according to manufacturer's instructions. Crude cell lysates were obtained by diluting 10^5 cells in 100 µL Tris-EDTA buffer (10 mM Tris-base,

1 mM EDTA, pH 8.0) supplemented with 200 µg/mL proteinase K. The mixtures were incubated at 56 °C for 30 minutes to lyse the cells, followed by 10 minute incubation at 95 °C to heat-inactive the proteinase K. Both the extracted genomic DNA and crude cell lysate were diluted 25x in the final LAMP-LUMID reaction (1 µL lysate in a 25 µL LAMP-LUMID reaction), using the same conditions as described above.

LAMP-LUMID with camera-based readout. The 3D-printed readout box was designed using Autodesk Phusion software and printed using PLA filament in a standard stereolithography 3D printer. Luminescence signal was recorded using a SONY DSC-RX100 digital camera, using an exposure time of 90 s, F value of 1.8 and ISO value of 6400. The camera was controlled by external Bluetooth remote for continuous shooting over 1 hour. RAW files were converted to JPG files using Sony Imaging Edge, and mean blue (B) and green (G) intensities per well were extracted from split RGB channels using a custom MATLAB (Mathworks) script (supplementary Figure S25). LAMP-LUMID reactions were performed as described above, using 1 µL of sample as input.

Clinical sample panel. Clinical panel consisted of 5x HPV16-positive, 5x HPV18-positive, 1x HPV16/18-double positive, 3x low-risk HPV-positive, 1x high-risk HPV51/52-positive and 2x HPV-negative DNA isolates from tissue biopsies. An extensive overview can be found in supplementary Table S4. DNA extraction from tissue was performed using the automated EMAG system (Biomerieux), according to manufacturer's instructions. All isolated were subsequently tested using LAMP-LUMID (see above) and the Anyplex HPV28 multiplex real-time PCR assay (Seegene) to obtain HPV subtyping and corresponding cycle threshold values, according to manufacturer's instructions.

Ethics statement

The samples used in this study were obtained as part of standard HPV testing and patients did not object to the use of remnant sample material for quality purposes and research. Local medical ethics review committee of Jeroen Bosch Hospital approved use of the samples for this study (nWMO METC Brabant - NW2025-46). The clinical samples were anonymous and used for an application in line with the reason for sample obtainment.

Supplementary note: production cost LAMP-LUMID

tsNL-sso7d production: 1 L scale – 1 mM IPTG – 50 μg/L kanamycin

LB Medium	25 g	€568.70 per 5 kg	€**2.80** per 25 g	**Roth**
IPTG	283.3 mg	€1,553.40 per 100 g	€**3.70** per 283.3 mg	**Roth**
Kanamycin	50 mg	€239.20 per 100 g	€**0.12** per 50 mg	**Roth**
Buffers	-	-	Negligible	
Ni-NTA Resin	4 mL, can be reused for ~10x		~ €**3.00** per batch	**Qiagen**
Strep-Tactin® resin	2 mL, can be reused for >10x		~ €**3.00** per batch	**Iba**
Consumables (plastics, desalting columns, filtration columns etc)	-	-	~ €**20.00**	**Roth, Cytiva, Merck, Eppendorf**
Quality controls	-	-	~ €**10.00**	
TOTAL			€**42.62** = ~ €0.10 per nmol	

1 L culture yields 12.5 mg protein = 430 nmol protein

Thiazole Orange – maleimide production: 100 μL scale – 20 mM AEM – 24 mM NHS-TO

1-(2-Aminoethyl)maleimide hydrochloride	2 μmol = 353 μgram	€92 per 100 mg	€**0.32** per 353 μgram	**Sigma**
NHS-Thiazole Orange	2.4 μmol = 1.21 mg	€474 per 5 mg	€**114.4** per 1.21 mg	**Biotium**
DMSO	100 μL	€220.40 per 2.5 L	Negligible	
Consumables	-	-	Negligible	
TOTAL			€**114.72** = ~ €57.4 per μmol	

100 μL scale reaction yields 2 μmol of TO-maleimide

tsLUMID production: 1 mL scale – 10 μM protein – 100 μM TO-maleimide

TO maleimide	100 nmol	€57.4 per μmol	€5.74 per 100 nmol	*Own production*
tsNL-sso7d	10 nmol	~ €0.10 per nmol	€1.00 per 10 nmol	*Own production*
Consumables (plastics, desalting columns, filtration columns etc)	-	-	~ €**20.00**	**Roth, Cytiva, Merck, Eppendorf**
TOTAL			€26.74 = €3.57 per nmol	

1 mL scale reaction yields 7.5 nmol tsLUMID sensor

LAMP-LUMD reaction: 25 µL scale – 10 nM tsLUMID – 1 µM bst esterase

Bst. 2.0 polymerase + buffers	8 U	€353 per 8000 U	€**0.35** per 8 U	**NEB**
dNTPs	35 nmol	€307 per 40 µmol	€**0.27** per 35 nmol	**NEB**
Bst esterase	25 pmol	€191 per 10 mg	€**0.01** per 25 pmol	**Sigma**
tsLUMID	250 fmol	€3.57 per nmol	< €**0.01** per 250 fmol	*Own production*
Primers	40 pmol	€115.22 per 100 nmol	€**0.09** per 40 pmol	**IDT**
Endurazine	0.25 µL	€11,781 per 10 mL	€**0.29** per 0.25 µL	**Promega**
Betaine	12.5 nmol	€494 per 2.5 kg	**negligible**	**Thermo Fisher**

LAMP-LUMID per-reaction costs: €1.02

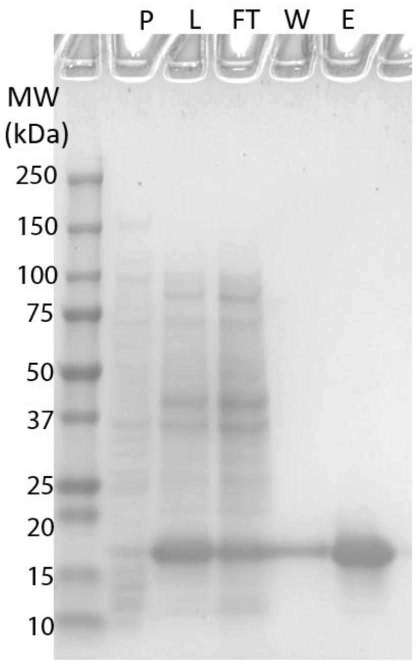

Supplementary Figure S1 | SDS-PAGE analysis of expression and purification of thermostable NanoLuc. Thermostable NanoLuc was expressed in *E. coli* and purified by using Ni^{2+} affinity chromatography. P: pellet, insoluble fraction of cell lysate, L: supernatant of cell lysate; FT/W/E: Ni^{2+} affinity chromatography flow through, wash and elution fractions. Marker: Precision Plus Protein™ marker (BioRad). The molecular weight of thermostable NanoLuc is 20.03 kDa.

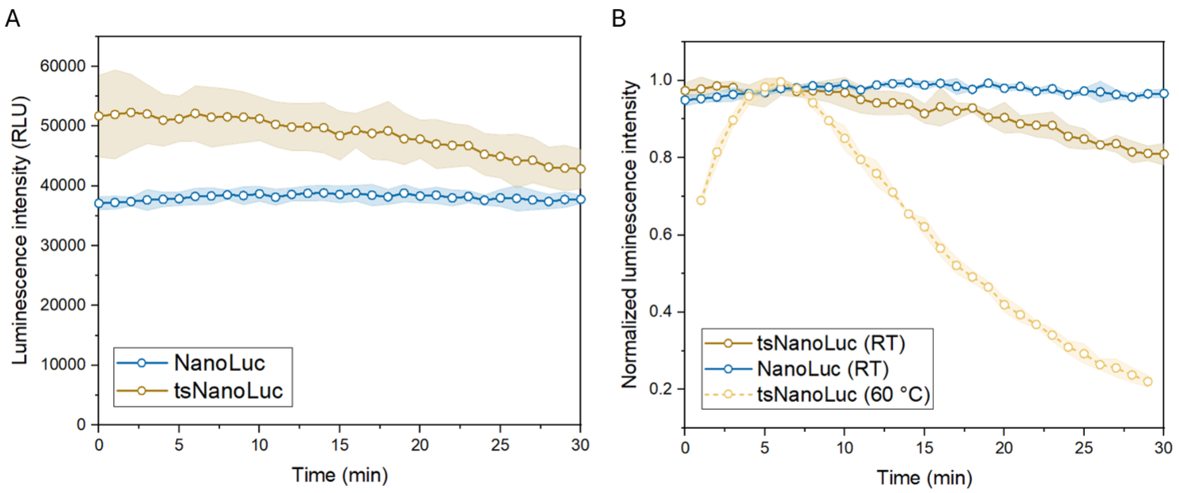

Supplementary Figure S2 | Comparison of luminescence intensity of NanoLuc, thermostable NanoLuc and thermostable NanoLuc at room temperature and 60 °C. A) Luminescence intensity (460 nm) of NanoLuc and Thermostable NanoLuc (tsNanoLuc) at room temperature. B) Normalized luminescence intensity (460 nm) of NanoLuc, tsNanoLuc at room temperature and tsNanoLuc at 60 °C. Please note that room temperature measurements were measured on a different plate reader (Tecan Spark) than 60-65 °C measurements (BMG labtech FLUOstar omega) and therefore absolute intensities cannot be compared. Assays were performed using 100 pM of luciferase protein and 1000x diluted Furimazine substrate, in 1xPBS + 1 mg/mL BSA at pH 7.4. Data represents mean ± sd, with n = 2 technical replicates.

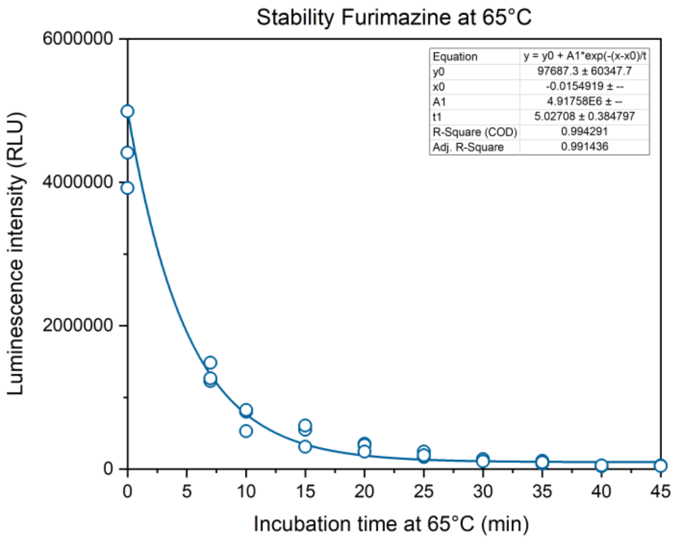

Supplementary Figure S3 | Stability of Furimazine substrate at LAMP reaction temperature. 500x diluted Furimazine substrate was incubated at 65 °C for 7 to 45 minutes and cooled down directly after incubation. After 45 minutes, the incubated Furimazine was combined in a 1:1 (v/v) ratio with 2 nM of thermostable NanoLuc and luminescence was recorded at 460 nm (RLU = relative light units). All reactions were performed in 1xPBS + 1 mg/mL BSA at pH 7.4. Circles represent individual data points, with n = 3 technical replicates. Line represents exponential decay function fitted to the data. Fitting parameters are indicated in the graph.

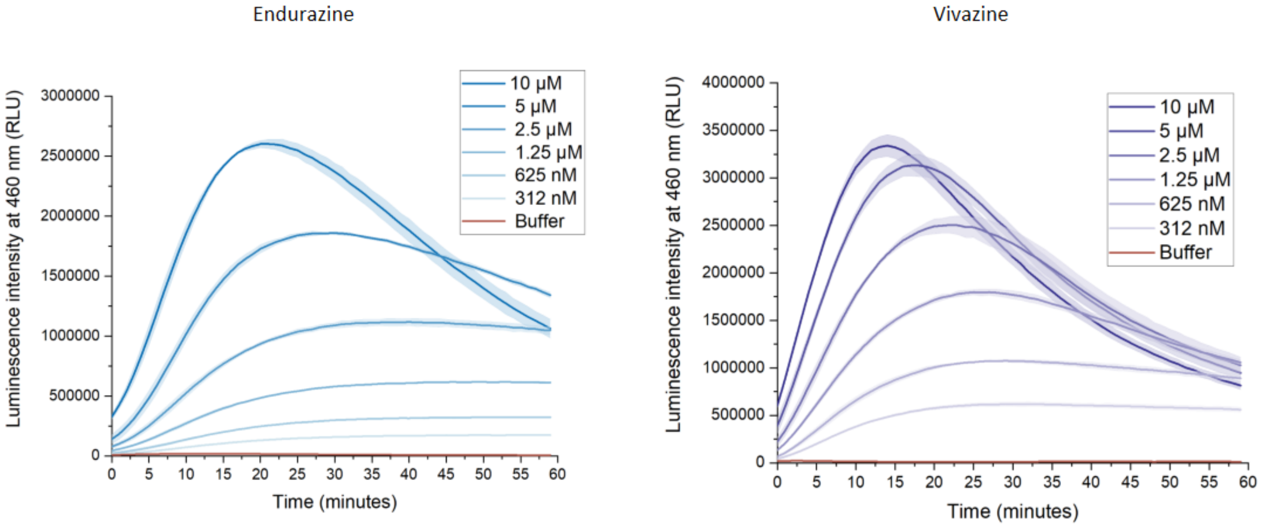

Supplementary Figure S4 | Comparison of esterase-mediated deprotection of Vivazine and Endurazine substrates. 312 nM – 10 µM of Bst. esterase was combined with 1 nM of tsLUMID and 100x diluted Endurazine (blue, left) or Vivazine (purple, right) substrate, in 1xPBS + 1 mg/mL BSA at pH 7.4. Luminescence was recorded at 460 nm (RLU = relative light units) for the duration of 1 hour at 40 °C. Data represents mean ± sd, with n = 3 technical replicates.

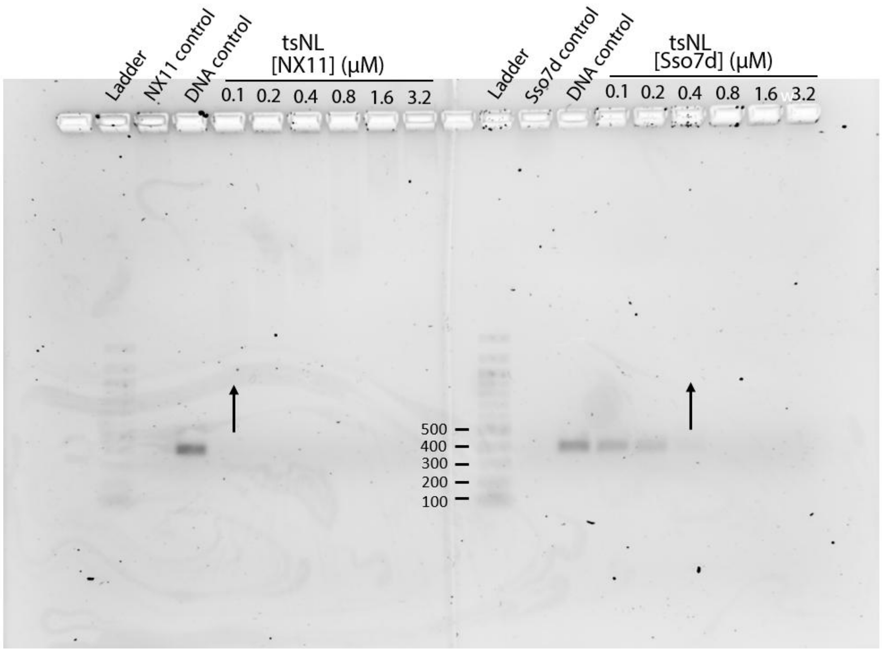

Supplementary Figure S5 | Electrophoretic mobility shift assay to compare NX11 and Sso7d dsDNA binding. A 383 bp DNA fragment (total [DNA] = 500 nM of basepairs) was incubated with 0.1-3.2 μM of tsNLuc-NX11/Sso7d fusion protein for 1.5 hours in 1xPBS. Reactions were combined with 5:1 with loading dye (containing no SDS) and immediately run for 45 min on a 1.5% agarose gel. Gel was stained for 15 min in a 1xTAE solution containing 1xdsDNA Green (LumiProbe) and destained for 15 min in demi water prior to imaging. Ladder: GeneRuler 100 bp ladder (ThermoFisher), protein controls: 3.2 μM of protein without DNA, DNA controls: 500 nM of DNA basepairs without protein.

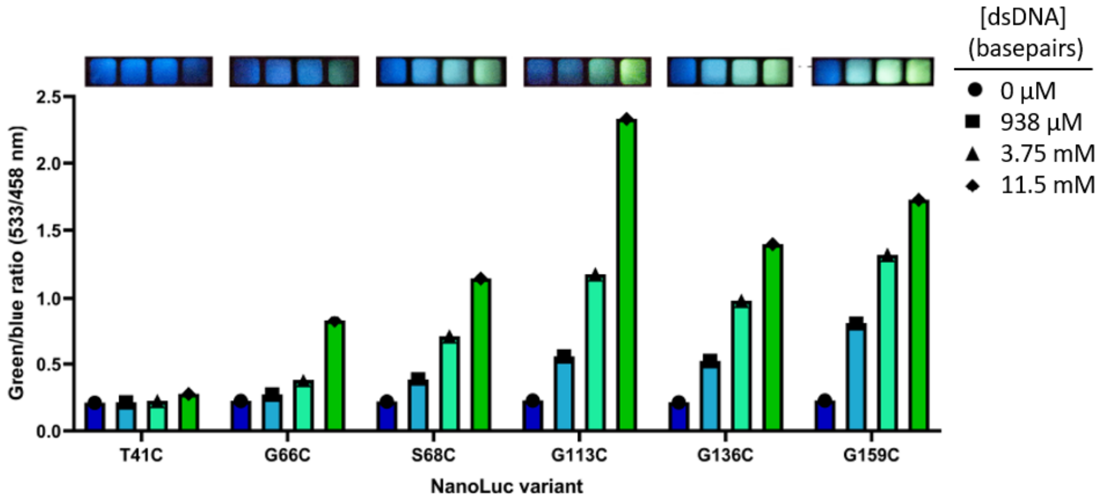

Supplementary Figure S6 | Pre-screening of dye positions within the NanoLuc luciferase without the presence of a DNA-binding protein. Dye conjugates were produced as described in methods 'Conjugation of maleimide-activated dyes to tsNLuc-NX11/Sso7d', using single-cysteine mutants (indicated at the X-axis) of the native NanoLuc luciferase. The conjugates (1 nM) were added to a 4-fold dilution series of Salmon Sperm DNA ranging from (in base pairs of DNA) 938 μM – 11.5 mM. Incubation was performed at room temperature for 30 minutes in 1xPBS + 1 mg/mL BSA, pH 7.4. Furimazine was added to a final dilution of 1000x and the luminescence was measured at 398-653 nm. The luminescent signal was also captured using a camera (Sony DSC-RX100) through a hole in a Styrofoam box to exclude the surrounding light. The photographs were taken with an exposure time of +/- 15 seconds, and ISO value of 3200. Date represents mean values, with n = 3 individual preparations of dsDNA.

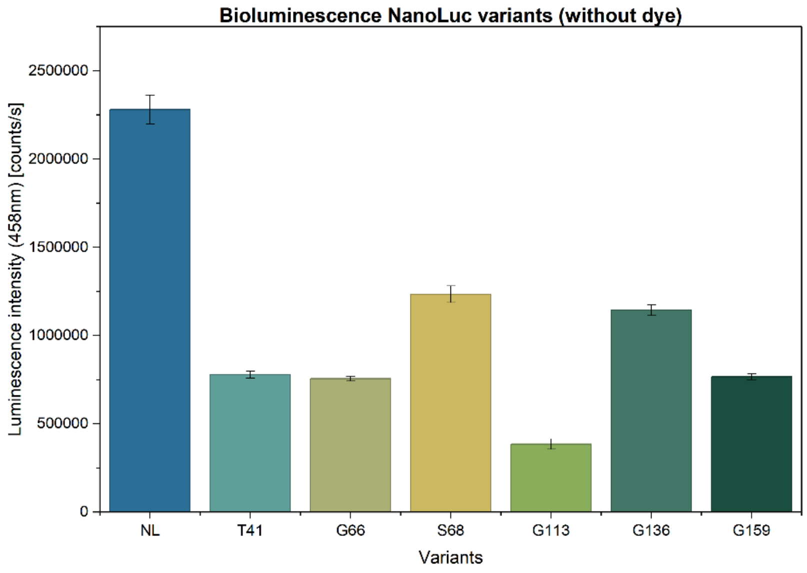

Supplementary Figure S7 | Effect of cysteine mutations on NanoLuc luciferase activity without the presence of the intercalating dye**.** The NanoLuc cysteine mutants (1 nM) were combined with Furimazine (1000x dilution) in 1xPBS + 1 mg/mL BSA. Immediately after, the luminescence was measured at 398-653 nm. Data represents mean ± standard deviation, with n = 3 technical replicates.

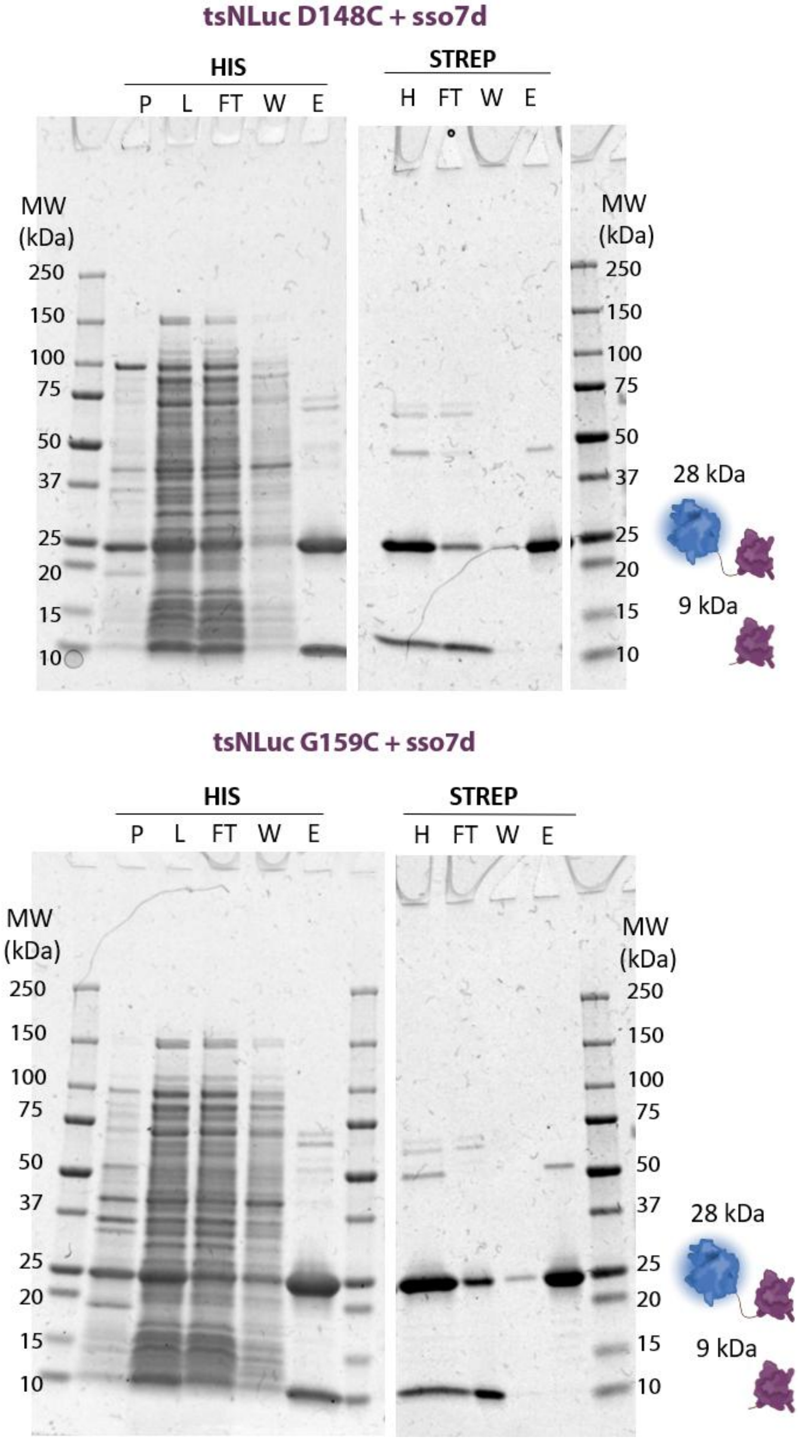

Supplementary Figure S8 | Expression and purification of tsNLuc-Sso7d fusion proteins. Fusion proteins of thermostable NanoLuc and Sso7d were expressed in *E. coli* and purified by using Ni^{2+} affinity chromatography 'HIS' followed by Strep-Tactin affinity chromatography 'STREP'. HIS) P: pellet, insoluble fraction of cell lysate, L: supernatant of cell lysate; FT/W/E: Ni^{2+} affinity chromatography flow through, wash and elution fraction. STREP) H: elution fraction of Ni^{2+} column, FT/W/E: Strep-Tactin affinity chromatography flow through, wash and elution fractions. Markers: Precision Plus Protein™ marker (BioRad). The molecular weight of tsNanoLuc-Sso7d fusions is ~ 29.2 kDa.

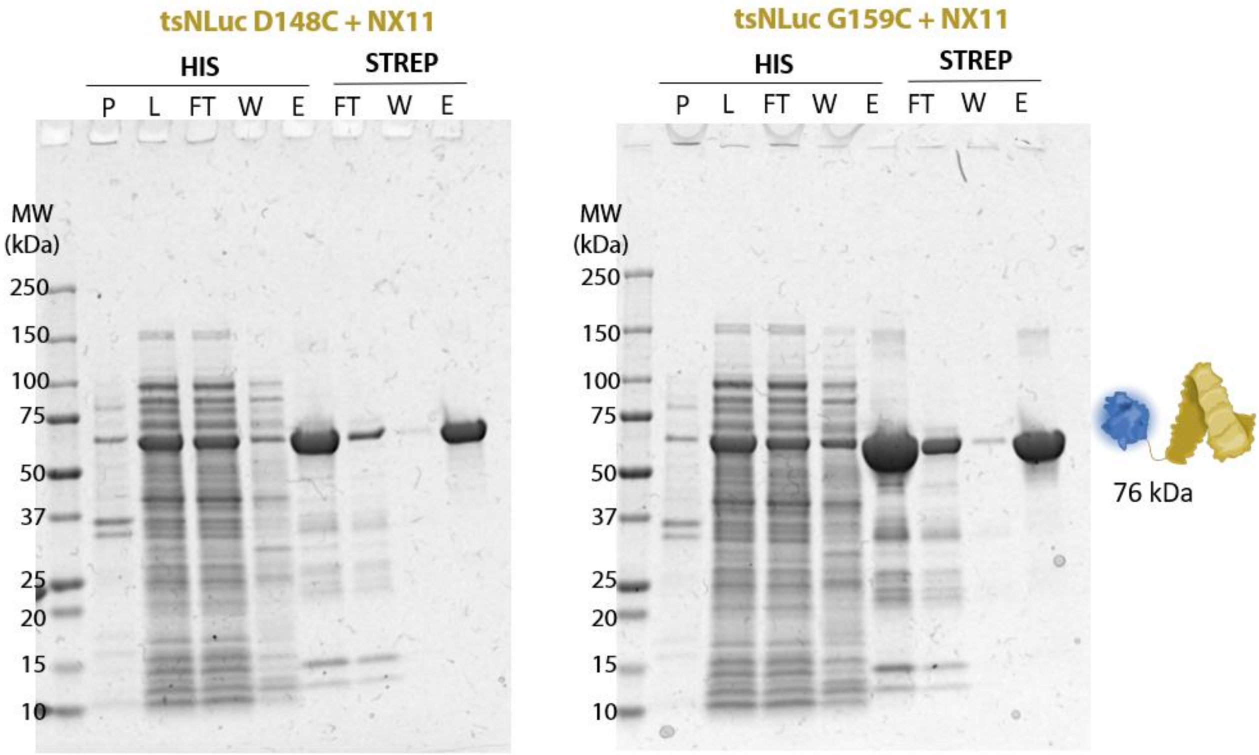

Supplementary Figure S9 | Expression and purification of tsNLuc-NX11 fusion proteins. Fusion proteins of thermostable NanoLuc and NX11 were expressed in *E. coli* and purified by using Ni^{2+} affinity chromatography 'HIS' followed by Strep-Tactin affinity chromatography 'STREP'. HIS) P: pellet, insoluble fraction of cell lysate, L: supernatant of cell lysate; FT/W/E: Ni^{2+} affinity chromatography flow through, wash and elution fraction. STREP) FT/W/E: Strep-Tactin affinity chromatography flow through, wash and elution fractions. Markers: Precision Plus Protein™ marker (BioRad). The molecular weight of tsNanoLuc-NX11 fusions is ~ 76.2 kDa.

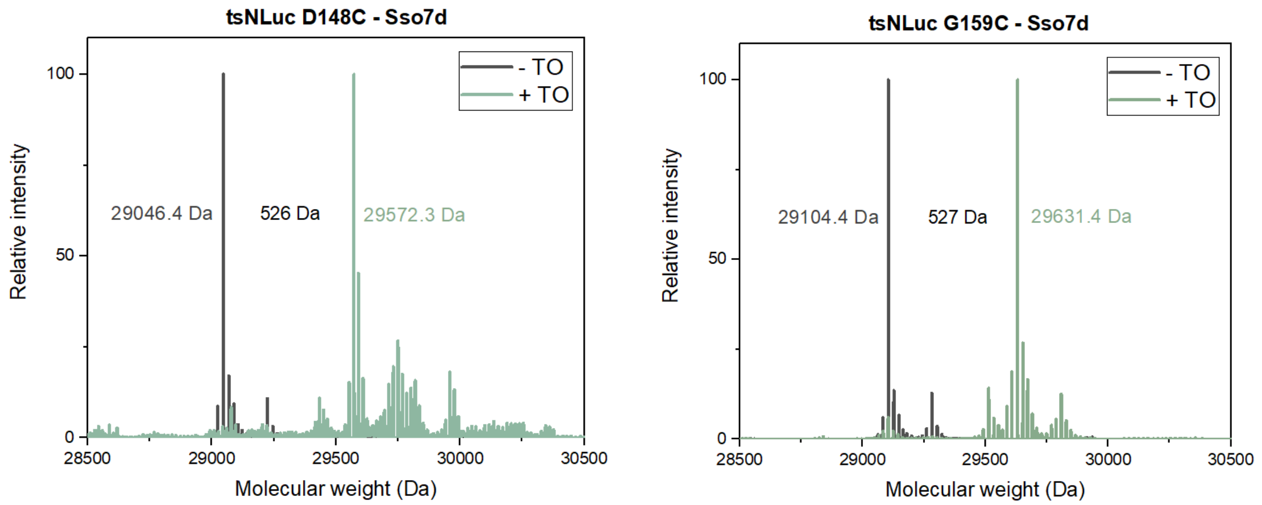

Supplementary Figure S10 | ESI-QToF mass spectra of tsNanoLuc-Sso7d fusion proteins, before (black) and after (green) dye coupling. Left: tsNanoLuc(D148C)-sso7d (calculated mass without dye: 29046.3 Da, with one dye: 29574.0 Da). Right: tsNanoLuc(G159C)-sso7d (calculated mass without dye: 29104.3 Da, with one dye: 29632.0 Da). Calculated molecular weight increase upon dye conjugation is 527.7 Da.

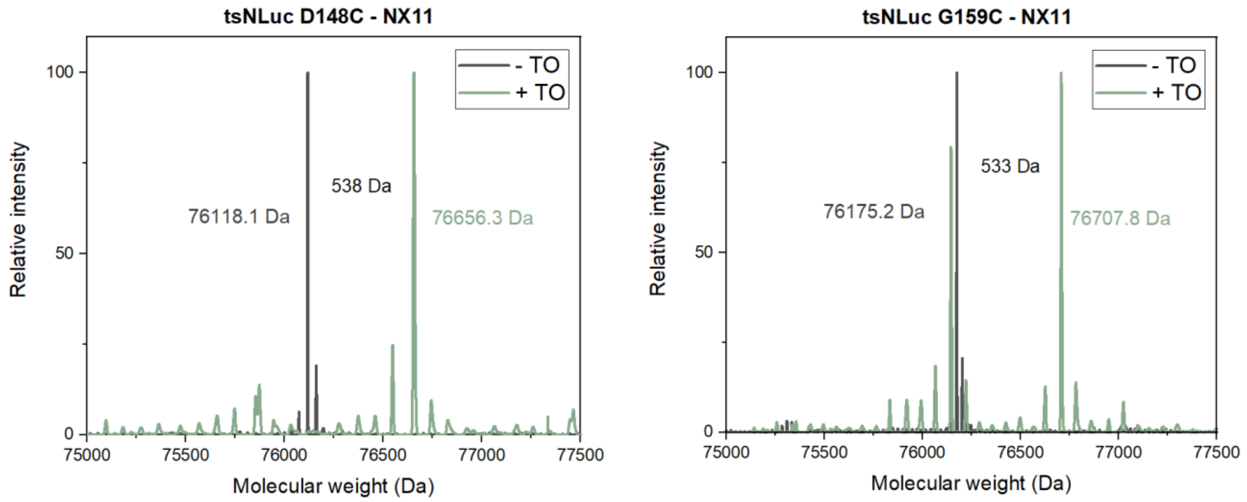

Supplementary Figure S11 | ESI-QToF mass spectra of tsNanoLuc-NX11 fusion proteins, before (black) and after (green) dye coupling. Left: tsNanoLuc(D148C)-NX11 (calculated mass without dye: 76118.0 Da, with one dye: 76645.2 Da). Right: tsNanoLuc(G159C)-sso7d (calculated mass without dye: 76176.0 Da, with one dye: 76703.2 Da). Calculated molecular weight increase upon dye conjugation is 527.7 Da. Please note that due to salt-adducts, we obtained low-resolution spectra with large error margins.

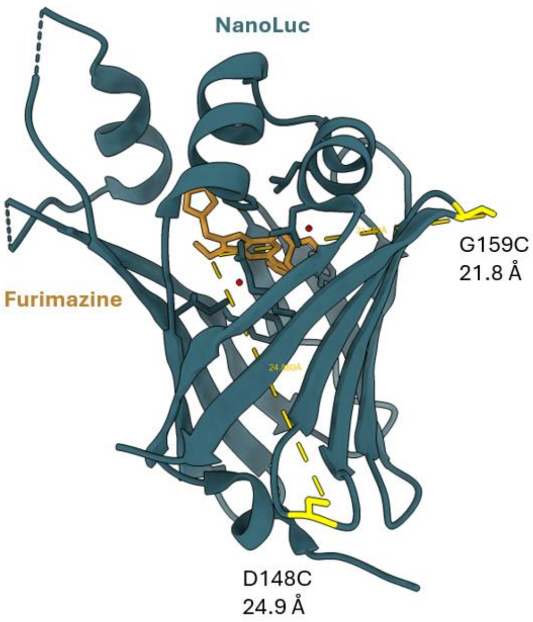

Supplementary Figure S12 | Schematic overview of NanoLuc structure in complex with azacoelenterazine substrate analog (in orange) in the catalytic site (PDB 8BO9) with cysteine mutations highlighted in yellow. Dashed lines indicate distance from the cysteine's thiol to the substrate's oxidation site.

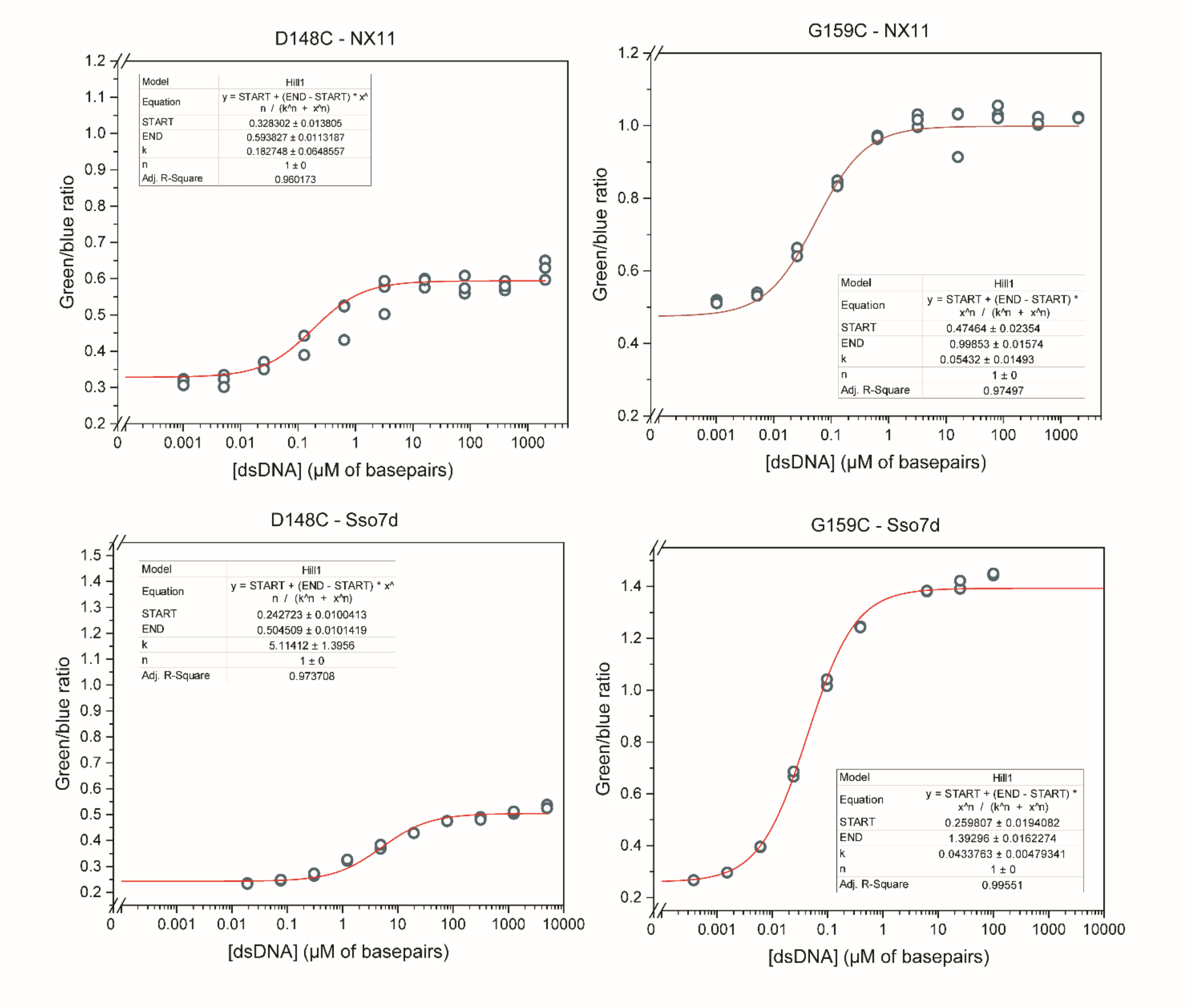

Supplementary Figure S13 | Model fits of different tsLUMID variants. The sensor response was measured in the presence of salmon sperm dsDNA ranging from 400 pM – 5 mM, depending on the affinity of the tested sensor. Experiments were performed in technical replicates with n = 2 (Sso7d) or n = 3 (NX11) independent preparations of the dsDNA, with 1 nM of sensor protein and 30 minutes of incubation. The acquired data was fitted with a Hill function with offset. Insets represent the fitting parameters and adjusted R-squared value. For all sensor variants, the Michaelis Menten constant (n) was fixed to 1 assuming no cooperativity. Circles represent individual data points and lines represent model fits.

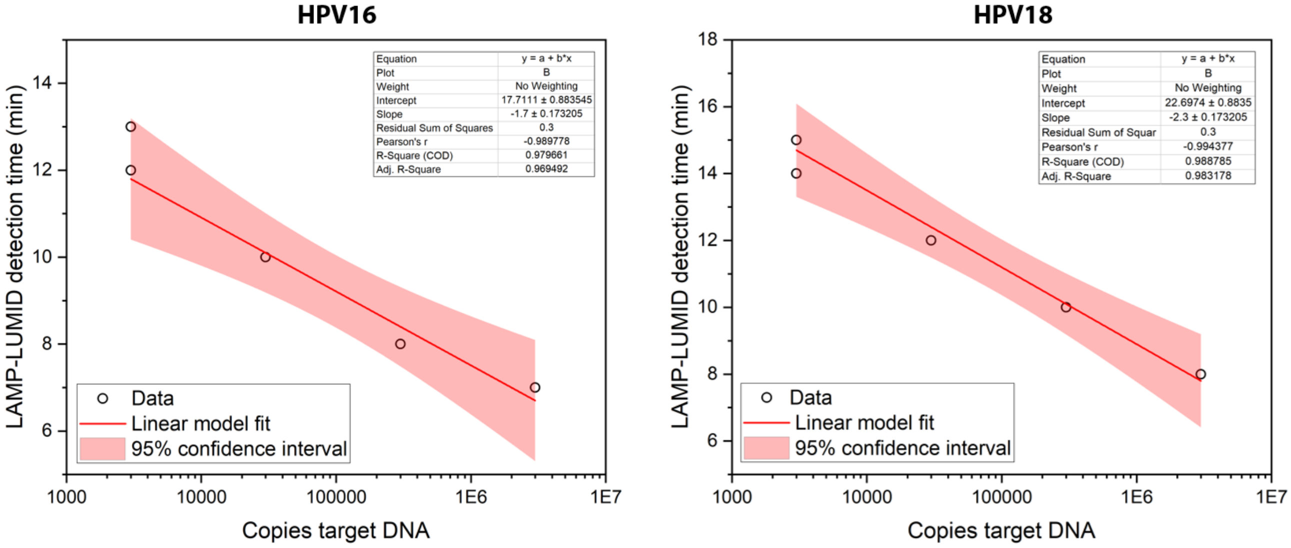

Supplementary Figure S14 | Log-linear correlation between input target concentrations and LAMP-LUMID detection time. The amount of input copies of target DNA (HPV16 left, HPV18 right) are plotted against the LAMP-LUMID detection time, which is defined as the time at which the green/blue ratio first surpasses the mean green/blue ratio + 8 times the standard deviation of all samples at t = 5 min. A linear regression model was fitted to the log-transformed data: $y = a + b^*(\log_{10}(x))$, with x = copies of target DNA and y = LAMP-LUMID detection time. Circles represent individual data points and red lines indicate model fit with 95% confidence bands. Exact fitting parameters are indicated in each graph.

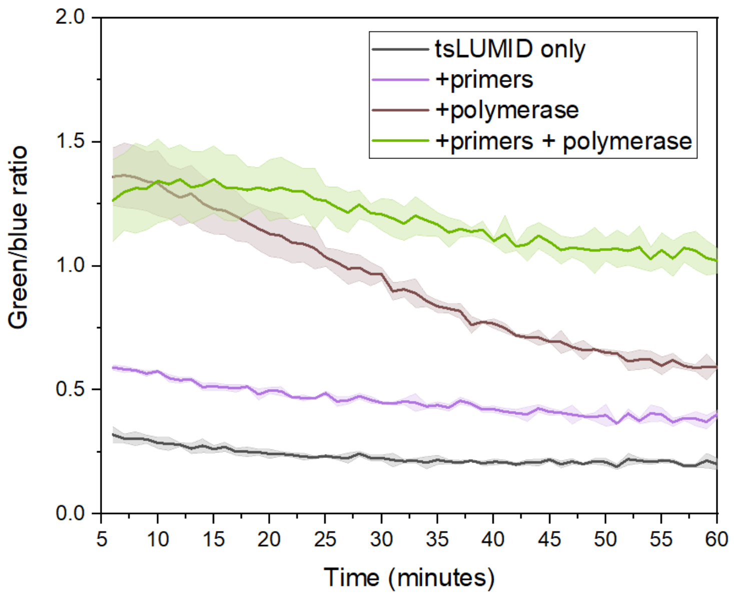

Supplementary Figure S15 | Background controls LAMP-LUMID. Controls were performed using standard HPV18 LAMP-LUMID assay conditions (see methods) without target DNA, exchanging different assay components with milliQ water. tsLUMID only (grey): no primers and no polymerase, +primers (pink): no polymerase, +polymerase (brown): no primers, +primer + polymerase (green): standard LAMP-LUMID conditions. Data represents mean ± sd, with n = 3 technical replicates.

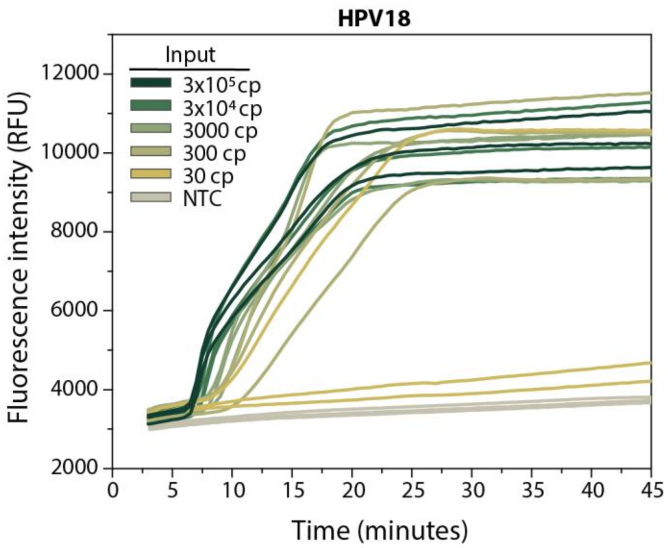

Supplementary Figure S16 | Fluorescent intercalating dye-based real-time LAMP targeting HPV18 E7 using synthetic genes as input. LAMP reactions were performed at 65 °C for 45 minutes with the addition of 300 nM dsDNA green (LumiProbe). Fluorescence intensity was monitored using the Bio-Rad CFX96 Touch Real-Time PCR Detection System. Lines represent individual reactions with n = 3 technical replicates per input concentration.

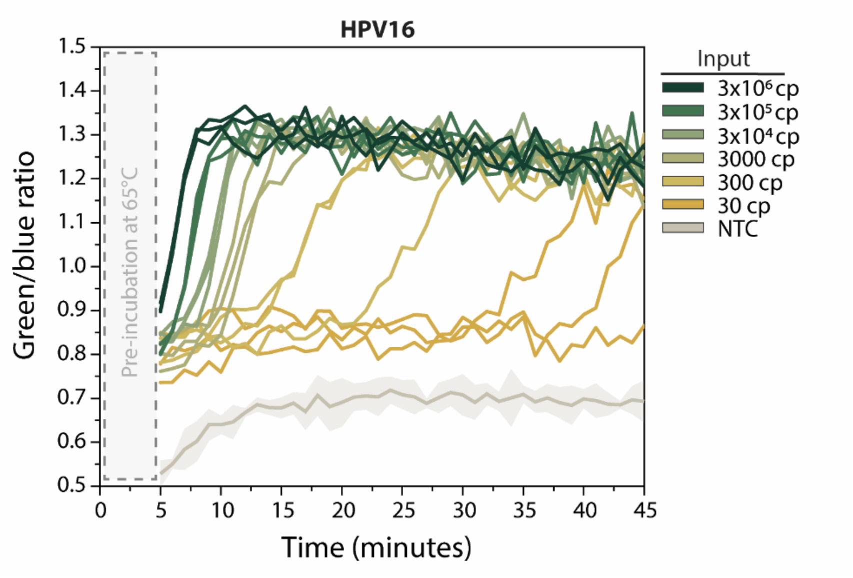

Supplementary Figure S17 | LAMP-LUMID assay targeting the HPV16 E7 gene using synthetic DNA fragments. Reactions were performed at 65 °C for 45 minutes with the addition of 5 nM tsLUMID, 1 μM esterase and 100x diluted Endurazine substrate. Reactions were incubated for 5 min at 65 °C prior to readout. Lines of negative reactions represent mean ± standard deviation with n = 3 technical replicates. Lines of other positive reactions represent individual reactions with n = 3 technical replicates.

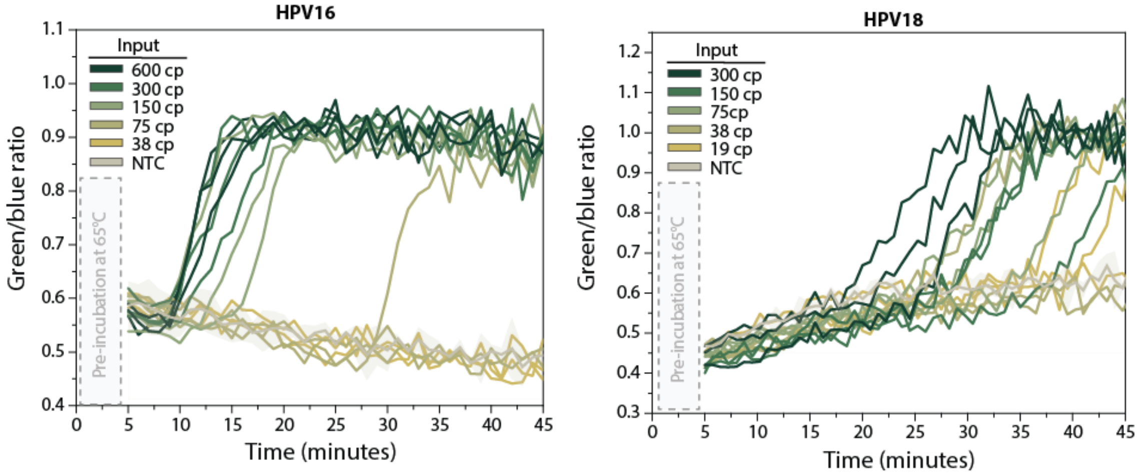

	HPV16	HPV18
**Input**	**Detected replicates**	**Detected replicates**
600 copies	*3/3*	*Not tested*
300 copies	*3/3*	*3/3*
150 copies	*3/3*	*3/3*
75 copies	*1/3*	*3/3*
37 copies	*0/3*	*1/3*
19 copies	*Not tested*	*2/3*

Supplementary Figure S18 | Real-time LAMP-LUMID targeting HPV16 (left) and HPV18 (right) E7 using synthetic genes as input for limit of detection (LOD) determination. LAMP reactions were performed at 65 °C for 45 minutes with the addition of 5 nM tsLUMID, 1 µM esterase and 100x diluted Endurazine substrate. Reactions were incubated for 5 min at 65 °C prior to readout. Lines represent individual reactions with n = 3 technical replicates per input concentration. Lines of negative reactions represent mean ± standard deviation with n = 3 technical replicates. Table displays an overview of the positive replicates per input concentration.

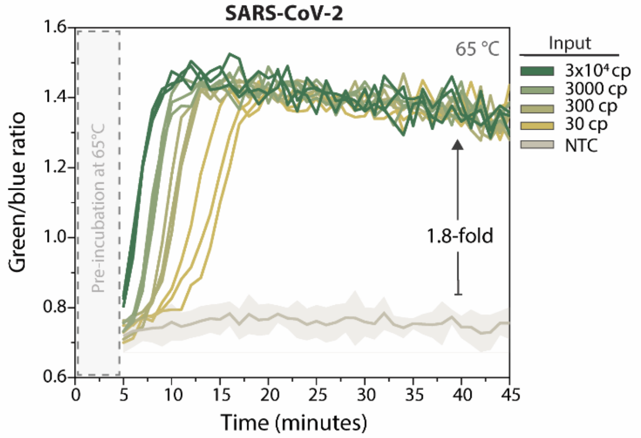

Supplementary Figure S19 | Real-time LAMP-LUMID targeting SARS-CoV-2 N-gene using synthetic genes. LAMP reactions were performed at 65 °C for 45 minutes with the addition of 5 nM tsLUMID, 1 µM esterase and 100x diluted Endurazine substrate. The SARS-CoV-2 primer set was taken from our previous work[27] and can be found in supplementary Table S3. Reactions were incubated for 5 min at 65 °C prior to readout. Lines represent individual reactions with n = 3 technical replicates per input concentration. Indicated fold-change represent the fold-change increase in green/blue ratio at t = 45 min.

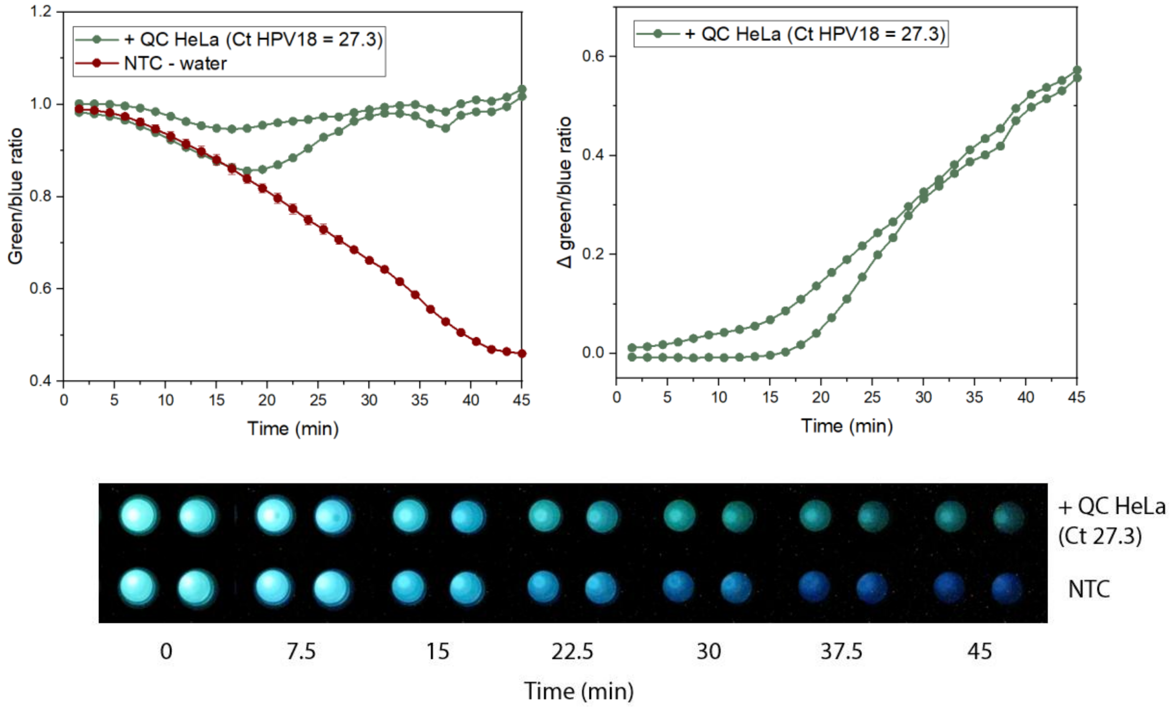

Supplementary Figure S20 | Camera-based LAMP-LUMID on quality control material using baseline subtraction. Analyzing the camera pictures results in decrease in green/blue ratio consistent with the decrease in signal intensity (left, bottom). Subtracting for this apparent decrease results in a response curve similar to plate reader measurements (right). Reactions were performed with 1 μL of quality control material (DNA isolates from HeLa cells, Ct HPV18 = 27.3) sample as input, using standard HPV18 LAMP conditions with the addition of 10 nM tsLUMID, 2 μM esterase, 100x diluted Endurazine in a single 25 μL reaction. For the positive reactions, line represent individual measurements with n = 2 technical replicates. For the water controls, lines represent mean ± standard deviation with n = 2 technical replicates.

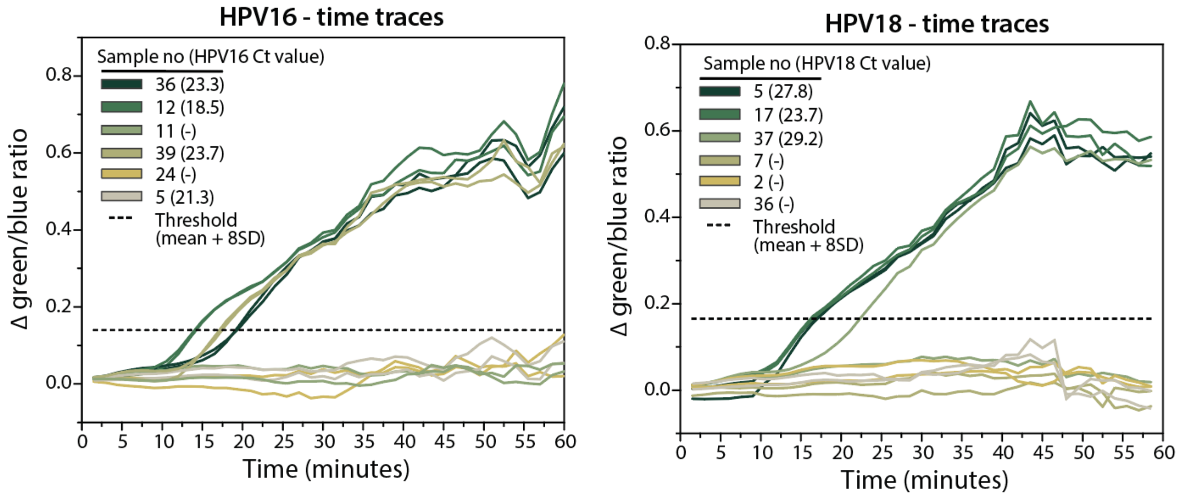

Supplementary Figure S21 | Green/blue traces over time for HPV16 (left) and HPV18 (right) extracted from camera pictures. Graphs shows traces of samples that were not included in main Figure 5. All reactions were performed with 1 μL of 10x diluted patient sample as input, using standard LAMP conditions with the addition of 10 nM tsLUMID, 2 μM esterase, 100x diluted Endurazine in a single 25 μL reaction. Lines represent technical replicates with n = 2.

```
atg gtg ttt acc ctg gaa gat ttt gtg ggc gat tgg gaa cag acc gcg gcg tat aac ctg
 M   V   F   T   L   E   D   F   V   G   D   W   E   Q   T   A   A   Y   N   L
gat cag gtg ctg gaa cag ggc ggc gtg agc agc ctg ctg cag aac ctg gcg gtg agc gtg
 D   Q   V   L   E   Q   G   G   V   S   S   L   L   Q   N   L   A   V   S   V
acc ccg att cag cgc att gtg cgc agc ggc gaa aac gcg ctg aaa att gat att cat gtg
 T   P   I   Q   R   I   V   R   S   G   E   N   A   L   K   I   D   I   H   V
att att ccg tat gaa ggc ctg agc gcg gat cag atg gcg cag att gaa gaa gtg ttt aaa
 I   I   P   Y   E   G   L   S   A   D   Q   M   A   Q   I   E   E   V   F   K
gtg gtg tat ccg gtg gat gat cat cat ttt aaa gtg att ctg ccg tat ggc acc ctg gtg
 V   V   Y   P   V   D   D   H   H   F   K   V   I   L   P   Y   G   T   L   V
att gat ggc gtg acc ccg aac atg ctg aac tat ttt ggc cgc ccg tat gaa ggc att gcg
 I   D   G   V   T   P   N   M   L   N   Y   F   G   R   P   Y   E   G   I   A
gtg ttt gat ggc aaa aaa att acc gtg acc ggc acc ctg tgg aac ggc aac aaa att att
 V   F   D   G   K   K   I   T   V   T   G   T   L   W   N   G   N   K   I   I
gat gaa cgc ctg att acc ccg gat ggc agc atg ctg ttt cgc gtg acc att aac ggc gtg
 D   E   R   L   I   T   P   D   G   S   M   L   F   R   V   T   I   N   G   V
agc ggc tgg cgc ctg ttt aaa aaa att agc ggt gga agc cat cac cac cat cac cat
 S   G   W   R   L   F   K   K   I   S   G   G   S   H   H   H   H   H   H
```

Supplementary Figure S22 | DNA and amino acid sequence of thermostable NanoLuc. Fusion of LargeBit and high-affinity SmallBit (yellow). A hexahistidine-tag (blue) is incorporated at the C- terminus.

```
atg ggt tgg tcc cat cct caa ttt gaa aaa atg gtg ttt acc ctg gaa gat ttt gtg ggc
 M   G   W   S   H   P   Q   F   E   K   M   V   F   T   L   E   D   F   V   G
gat tgg gaa cag acc gcg gcg tat aac ctg gat cag gtg ctg gaa cag ggc ggc gtg agc
 D   W   E   Q   T   A   A   Y   N   L   D   Q   V   L   E   Q   G   G   V   S
agc ctg ctg cag aac ctg gcg gtg agc gtg acc ccg att cag cgc att gtg cgc agc ggc
 S   L   L   Q   N   L   A   V   S   V   T   P   I   Q   R   I   V   R   S   G
gaa aac gcg ctg aaa att gat att cat gtg att att ccg tat gaa ggc ctg agc gcg gat
 E   N   A   L   K   I   D   I   H   V   I   I   P   Y   E   G   L   S   A   D
cag atg gcg cag att gaa gaa gtg ttt aaa gtg gtg tat ccg gtg gat gat cat cat ttt
 Q   M   A   Q   I   E   E   V   F   K   V   V   Y   P   V   D   D   H   H   F
aaa gtg att ctg ccg tat ggc acc ctg gtg att gat ggc gtg acc ccg aac atg ctg aac
 K   V   I   L   P   Y   G   T   L   V   I   D   G   V   T   P   N   M   L   N
tat ttt ggc cgc ccg tat gaa ggc att gcg gtg ttt gat ggc aaa aaa att acc gtg acc
 Y   F   G   R   P   Y   E   G   I   A   V   F   D   G   K   K   I   T   V   T
ggc acc ctg tgg aac ggc aac aaa att att gat gaa cgc ctg att acc ccg gat ggc agc
 G   T   L   W   N   G   N   K   I   I   D   E   R   L   I   T   P   D   G   S
atg ctg ttt cgc gtg acc att aac ggc gtg agc ggc tgg cgc ctg ttt aaa aaa att agc
 M   L   F   R   V   T   I   N   G   V   S   G   W   R   L   F   K   K   I   S
ggg ggt agc ggc ggc tcg ggg ggt agt ggt gga agc gca act gtg aaa ttt aaa tac aaa
 G   G   S   G   G   S   G   G   S   G   G   S   A   T   V   K   F   K   Y   K
ggg gaa gag aag gaa gta gat att agt aaa atc aaa aaa gtc tgg cgt gtt ggt aag atg
 G   E   E   K   E   V   D   I   S   K   I   K   K   V   W   R   V   G   K   M
att agc ttc acc tat gat gaa ggc gga ggc aaa acg ggt cgc ggc gcc gtg tcc gaa aaa
 I   S   F   T   Y   D   E   G   G   G   K   T   G   R   G   A   V   S   E   K
gac gcg ccg aaa gaa tta ctg cag atg ctg gag aaa caa aag aaa ggt gga agc cat cac
 D   A   P   K   E   L   L   Q   M   L   E   K   Q   K   K   G   G   S   H   H
cac cat cac cat
 H   H   H   H
```

Supplementary Figure S23 | DNA and amino acid sequence of thermostable NanoLuc and Sso7d fusion proteins. Fusion of thermostable NanoLuc and Sso7d through a 4x-GGS linker (yellow). A strep-tag (pink) and hexahistidine-tag (blue) is incorporated at the N- and C- terminus, respectively. The positions D148 (red) and G159 (green) are used for conjugation to Thiazole Orange.

atg ggt tgg tcc cat cct caa ttt gaa aaa atg gtg ttt acc ctg gaa gat ttt gtg ggc
M G W S H P Q F E K M V F T L E D F V G
gat tgg gaa cag acc gcg gcg tat aac ctg gat cag gtg ctg gaa cag ggc ggc gtg agc
D W E Q T A A Y N L D Q V L E Q G G V S
agc ctg ctg cag aac ctg gcg gtg agc gtg acc ccg att cag cgc att gtg cgc agc ggc
S L L Q N L A V S V T P I Q R I V R S G
gaa aac gcg ctg aaa att gat att cat gtg att att ccg tat gaa ggc ctg agc gcg gat
E N A L K I D I H V I I P Y E G L S A D
cag atg gcg cag att gaa gaa gtg ttt aaa gtg gtg tat ccg gtg gat gat cat cat ttt
Q M A Q I E E V F K V V Y P V D D H H F
aaa gtg att ctg ccg tat ggc acc ctg gtg att gat ggc gtg acc ccg aac atg ctg aac
K V I L P Y G T L V I D G V T P N M L N
tat ttt ggc cgc ccg tat gaa ggc att gcg gtg ttt gat ggc aaa aaa att acc gtg acc
Y F G R P Y E G I A V F D G K K I T V T
ggc acc ctg tgg aac ggc aac aaa att att gat gaa cgc ctg att acc ccg gat ggc agc
G T L W N G N K I I D E R L I T P D G S
atg ctg ttt cgc gtg acc att aac ggc gtg agc ggc tgg cgc ctg ttt aaa aaa att agc
M L F R V T I N G V S G W R L F K K I S
ggg ggt agc ggc ggc tcg ggg ggt agt ggt gga agc atg gtg gat tta cgc acc ctg ggc
G G S G G S G G S G G S M V D L R T L G
tat tct caa cag cag caa gag aag att aag ccg aag gtc cgc tcg acg gtg gct caa cat
Y S Q Q Q Q E K I K P K V R S T V A Q H
cat gag gcg ttg gtg ggt cac ggc ttt act cat gcg cat att gtt gcg ctc agt caa cat
H E A L V G H G F T H A H I V A L S Q H
cca gcg gca ctg ggc acc gtg gcg gtg aaa tat caa gat atg atc gcc gcg ctg ccg gag
P A A L G T V A V K Y Q D M I A A L P E
gcc acg cac gaa gcg atc gta ggc gtt ggc aaa cag tgg tct ggc gca cgt gcc ctg gag
A T H E A I V G V G K Q W S G A R A L E
gcg ctg ctt acc gtc gcg ggt gaa ttg cgt ggg ccg ccg ctg caa ttg gat acg ggg caa
A L L T V A G E L R G P P L Q L D T G Q
tta ctg aaa att gca aaa cgc ggc ggc gtg acc gcg gtt gaa gcc gtt cac gca tgg cgt
L L K I A K R G G V T A V E A V H A W R
aat gcc ctc acc ggt gcc ccg ctg aat ctc acg ccg gat cag gtc gtg gcc att gcc aag
N A L T G A P L N L T P D Q V V A I A K
cgc ggt ggt aaa cag gca ctc gaa acc gtt cag cgt ctg ctg ccg gaa ctg acc cag aaa
R G G K Q A L E T V Q R L L P E L T Q K
cac ggt ctg aca cca gat cag gtg gtg gcc atc gcc aaa cgc ggt ggt aaa caa gcc ctt
H G L T P D Q V V A I A K R G G K Q A L
gag act gtt cag cgg ctg ctc ccg gaa ttg acc cag aag cac gga ttg acc ccg gat caa
E T V Q R L L P E L T Q K H G L T P D Q
gtc gtc gcc att gca aag cgc ggc ggt aaa cag gca ctt gaa act gtg cag cgc ctg ctc
V V A I A K R G G K Q A L E T V Q R L L
cct gaa ctg acg cag aag cat ggt ctg acg ccg gac cag gtg gtc gct att gcc aag cgt
P E L T Q K H G L T P D Q V V A I A K R
ggg ggt aag cag gct ctt gaa acc gtc cag cgt ctg ctt ccg gaa tta act cag aaa cat
G G K Q A L E T V Q R L L P E L T Q K H
ggg ctg acg cca gac cag gtt gtg gca atc gcg aaa cgg ggc ggt aag caa gcg ctg gaa
G L T P D Q V V A I A K R G G K Q A L E
acc gtg caa cgt tta ttg ccg gag ctg aca cag aag cat ggc ctg act ccg gat cag gta
T V Q R L L P E L T Q K H G L T P D Q V
gtt gcg att gcg aaa cgc ggc ggc aag cag gcc ttg gaa acc gtt cag cgc ctc ctg cca
V A I A K R G G K Q A L E T V Q R L L P
gag ttg acg cag aaa cac ggt tta acg cca gat cag gtg gtg gcc atc gcc aaa cgc ggt
E L T Q K H G L T P D Q V V A I A K R G
ggt aaa caa gcc ctt gag act gtt cag cgg ctg ctc ccg gaa ttg acc cag aag cac gga
G K Q A L E T V Q R L L P E L T Q K H G
ttg acc ccg gat caa gtc gtc gcc att gca aag cgc ggc ggt aaa cag gca ctt gaa act
L T P D Q V V A I A K R G G K Q A L E T
gtg cag cgc ctg ctc cct gaa ctg acg cag aag cat ggt ctg acg ccg gac cag gtg gtc
V Q R L L P E L T Q K H G L T P D Q V V
gct att gcc aag cgt ggg ggt aag cag gct ctt gaa acc gtc cag cgt ctg ctt ccg gaa

```
 A   I   A   K   R   G   G   K   Q   A   L   E   T   V   Q   R   L   L   P   E
tta act cag aaa cat ggg ctg acg cca gac cag gtt gtg gca atc gcg aaa cgg ggc ggt
 L   T   Q   K   H   G   L   T   P   D   Q   V   V   A   I   A   K   R   G   G
aag caa gcg ctg gaa acc gtg caa cgt tta ttg ccg gag ctg aca cag aag cat ggc ctg
 K   Q   A   L   E   T   V   Q   R   L   L   P   E   L   T   Q   K   H   G   L
act ccg gat cag gta gtt gcg att gcg aaa cgc ggc ggc aag cag gcc ttg gaa acc gtt
 T   P   D   Q   V   V   A   I   A   K   R   G   G   K   Q   A   L   E   T   V
cag cgc ctc ctg cca gag ttg acg cag aaa cac ggt ttt gac ctc gag cac cac cac cac
 Q   R   L   L   P   E   L   T   Q   K   H   G   F   D   L   E   H   H   H   H
cac cac
 H   H
```

Supplementary Figure S24 | DNA and amino acid sequence of thermostable NanoLuc and NucleoX11 fusion proteins. Fusion of thermostable NanoLuc and NucleoX11 through a 4x-GGS linker (yellow). NucleoX11 consists of the native TALE N-terminus (grey) and 11 NucleoX repeats (1 repeated indicated in aqua) A strep-tag (pink) and hexahistidine-tag (blue) is incorporated at the N- and C- terminus, respectively. The positions D148 (red) and G159 (green) are used for conjugation to Thiazole Orange.

```
clc;        % Clear the command window.

close all;  % Close all figures
clear all;  % Erase all existing variables
workspace;  % Make sure the workspace panel is showing.

% extraction all photo names from selected folder
jpgFiles = dir('*.jpg'); %.jpg can be changed to any file type
numFiles = length(jpgFiles);

% reading in all RGB images
for k = 1:numFiles
    mydata{k} = imread(jpgFiles(k).name);
end

% use first image to extract circel coordinates
for k = 1
    subplot(2, 1, 1)
    imshow(mydata{k});% Get the gray image
    grayImage = im2gray(mydata{k});

    % enhance contrast
    contrastImage = adapthisteq(grayImage);

    % Remove noise
    filteredImage = medfilt2(contrastImage);
    n = 5; %filter steps
    for i = 1:n
        filteredImage = medfilt2(filteredImage);
    end

    % Get the binary image
    binaryImage = im2bw(filteredImage, 0.30);

    % dispaly binary image
    subplot(2, 1, 2);
    imshow(binaryImage);

    % find and visualize circles
```

```
    stats = regionprops('table', binaryImage, 'Centroid', 'Eccentricity',
'EquivDiameter');
    stats(stats.EquivDiameter < 120, : ) = [];

    statsArray = table2array(stats);

    centers = statsArray(:,1:2);
    diameter = statsArray(:,4);
    radii = diameter/3;

    viscircles(centers, radii,'EdgeColor','r');
end


% extracting g/b ratios from all images and all wells
GBcombined = [];
for k = 1:numFiles
    G = mydata{k}(:,:,2);
    B = mydata{k}(:,:,3);
    for n = 1: length(radii)
        mask = circles2mask(centers(n,1:2),radii(n,1),size(binaryImage));
        mean_G = mean(G(mask));
        mean_B = mean(B(mask));
        GBvalue = mean_G/mean_B;
        GBcombined = [GBcombined,GBvalue];
    end
end

% Combine all G/B values in array and add X,Y coordinates of wells
 GBfinal = reshape(GBcombined, length(radii), []);

 GBfinal(:,numFiles+1) = centers(:,1);
 GBfinal(:,numFiles+2) = centers(:,2);
```

Supplementary Figure S25 | MatLab script to extract green-over-blue ratios from camera pictures

Supplementary Table S1 | Mutagenesis primers to introduce single-cysteine mutations in tsNanoLuc and tsNanoLuc-DNA binding protein fusions.

Construct	Short description	Sequence (5' → 3')
tsNL(D148C)	Used for mutating the native aspartic acid at position 148 acid to a cysteine	GAACGCCTGATTACCCCGTGTGGC<br>AGCATGCTGTTC (forward)<br><br>GAAACAGCATGCTGCCACACGGGG<br>TAATCAGGCGTTC (reverse)
tsNL(G159C)	Used for mutating the native glycine at position 159 acid to a cysteine	CGTGACCATTAACTGCGTGAGCGG<br>CTGGC (forward)<br><br>GCCAGCCGCTCACGCAGTTAATGG<br>TCACG (reverse)

Supplementary Table S2 | HPV16 E7, HPV18 E7 and Sars-CoV-2 N synthetic genes. Sequences correspond to PCR fragments used for initial assay development.

Construct	Sequence (5' → 3')
HPV16 E7	GTCGGTGGACCGGTCGATGTATGTCTTGTTGCAGATCATCAAGAACACGTAG<br>AGAAACCCAGCTGTAATCATGCATGGAGATACACCTACATTGCATGAATATAT<br>GTTAGATTTGCAACCAGAGACAACTGATCTCTACTGTTATGAGCAATTAAATG<br>ACAGCTCAGAGGAGGAGGATGAAATAGATGGTCCAGCTGGACAAGCAGAAC<br>CGGACAGAGCCCATTACAATATTGTAACCTTTTGTTGCAAGTGTGACTCTACG<br>CTTCGGTTGTGCGTACAAAGCACACACGTAGACATTCGTACTTTGGAAGACC<br>TGTTAATGGGCACACTAGGAATTGTGTGCCCCATCTGTTCTCAGAAACCATA<br>ATCTACCATGGCTGATCCTGCAGGTACCAATGGGGAAGAGGGTACGGGATG<br>TAATGGATGGTTTTATGTAGAGGCTGTAGTGGAAAAAAAAACAGGGGATGCT<br>ATATCAGATGACGAGAACGAAAATGACAGTGATACAGGTGAAGATTTGGTAG<br>ATTTTATAGTAAATGATAATGATTATTTAACACAGGCAGAAACAGAGACAGCA<br>CATGC
HPV18 E7	TATGCATGGACCTAAGGCAACATTGCAAGACATTGTATTGCATTTAGAGCCC<br>CAAAATGAAATTCCGGTTGACCTTCTATGTCACGAGCAATTAAGCGACTCAG<br>AGGAAGAAAACGATGAAATAGATGGAGTTAATCATCAACATTTACCAGCCCG<br>ACGAGCCGAACCACAACGTCACACAATGTTGTGTATGTGTTGTAAGTGTGAA<br>GCCAGAATTGAGCTAGTAGTAGAAAGCTCAGCAGACGACCTTCGAGCATTCC<br>AGCAGCTGTTTCTGAACACCCTGTCCTTTGTGTGTCCGTGGTGTGCATCCCA<br>GCAGTAAGCAACAATGGCTGATCCAGAAGGTACAGACGGGGAGGGCACGG<br>GTTGTAACG
SARS-CoV-2 N-gene	TAATACGACTCACTATAGGGATGTCTGATAATGGACCCCAAAATCAGCGAAATGCACCC<br>CGCATTACGTTTGGTGGACCCTCAGATTCAACTGGCAGTAACCAGAATGGAGAACGCA<br>GTGGGGCGCGATCAAAACAACGTCGGCCCCAAGGTTTACCCAATAATACTGCGTCTTG<br>GTTCACCGCTCTCACTCAACATGGCAAGGAAGACCTTAAATTCCCTCGAGGACAAGGC<br>GTTCCAATTAACACCAATAGCAGTCCAGATGACCAAATTGGCTACTACCGAAGAGCTAC<br>CAGACGAATTCGTGGTGGTGACGGTAAAATGAAAGATCTCAGTCCAAGATGGTATTTCT<br>ACTACCTAGGAACTGGGCCAGAAGCTGGACTTCCCTATGGTGCTAACAAAGACGGCAT<br>CATATGGGTTGCAACTGAGGGAGCCTTGAATACACCAAAAGATCACATTGGCACCCGC<br>AATCCTGCTAACAATGCTGCAATCGTGCTACAACTTCCTCAAGGAACAACATTGCCAAA<br>AGGCTTCTACGCAGAAGGGAGCAGAGGCGGCAGTCAAGCCTCTTCTCGTTCCTCATC<br>ACGTAGTCGCAACAGTTCAAGAAATTCAACTCCAGGCAGCAGTAGGGGAACTTCTCCT<br>GCTAGAATGGCTGGCAATGGCGGTGATGCTGCTCTTGCTTTGCTGCTGCTTGACAGATT<br>GAACCAGCTTGAGAGCAAAATGTCTGGTAAAGGCCAACAACAACAAGGCCAAACTGTC

	ACTAAGAAATCTGCTGCTGAGGCTTCTAAGAAGCCTCGGCAAAAACGTACTGCCACTA AAGCATACAATGTAACACAAGCTTTCGGCAGACGTGGTCCAGAACAAACCCAAGGAAA TTTTGGGGACCAGGAACTAATCAGACAAGGAACTGATTACAAACATTGGCCGCAAATTG CACAATTTGCCCCCAGCGCTTCAGCGTTCTTCGGAATGTCGCGCATTGGCATGGAAGT CACACCTTCGGGAACGTGGTTGACCTACACAGGTGCCATCAAATTGGATGACAAAGAT CCAAATTTCAAAGATCAAGTCATTTTGCTGAATAAGCATATTGACGCATACAAAACATTCC CACCAACAGAGCTTCGCCTAGGCCGCTGAGCAATAACTAGCATAACCCCTTGGGGCC TCTAAACGGGTCTTGAGGGGTTTTTTGCTGAAAACCTCGCTCGCTGAGGTGTCAATCGT CGGAGCCGCTGAGCAATAACTAGCATAACCCCTTGGGGCCTCTAAACGGGTCTTGAG GGGTTTTTTGCATGGTCATAGCTGTTTCCTG

Supplementary Table S3 | LAMP primer sets targeting HPV16 E7, HPV18 E7 and Sars-CoV-2 N-gene

Primer name	Sequence (5' → 3')
HPV16	
Forward inner primer	TGGGGCACACAATTCCTAGTCACACAGTAGACATTCGT
Backward inner primer	TCAGAAACCATAATCTACCATGGCATTACATCCCGTACCCTCTT
Forward outer primer	TCGGTTGTGCGTACAAAG
Backward outer primer	AGCCTCTACATAAAACCATCC
Forward loop	CCCATTAACAGGTCTTCCAAAGT
Backward loop	CCTGCAGGTACCAATGGGG
HPV18	
Forward inner primer	GCTTCACACTTACAACACATACACAATCAACATTTACCAGCCCG
Backward inner primer	TTGAGCTAGTAGTAGAAAGCTCAGCACGGACACACAAAGGACA
Forward outer primer	TCAGAGGAAGAAAACGATGA
Backward outer primer	GTTGCTTACTGCTGGGAT
Forward loop	ACGTTGTGGTTCGGCTCGT
Backward loop	GCATTCCAGCAGCTGTTTCTGAAC
SARS-CoV-2	
Forward inner primer	TCCCCTACTGCTGCCTGGAGGCAGTCAAGCCTCTTCTCG
Backward inner primer	TCTCCTGCTAGAATGGCTGGCATCTGTCAAGCAGCAGCAAAG
Forward outer primer	GCCAAAAGGCTTCTACGCA
Backward outer primer	TTGCTCTCAAGCTGGTTCAA
Forward loop	GCGACTACGTGATGAGGAA
Backward loop	GGCGGTGATGCTGCTCTT

Supplementary Table S4 | Overview clinical samples with HPV subtype determination, corresponding qPCR Ct values and LAMP-LUMID threshold time (Tt). Internal control (IC) is used to confirm presence of human cellular material. n.d. = not detected, n.t. = not tested.

**HPV 16+ samples**								
Panel no	# subtypes	HPV subtype	Ct value HPV16	Ct value HPV18	Ct values (other HPV subtypes	Ct value internal control	HPV16 LAMP-LUMID Tt (replicate 1, replicate 2)	HPV18 LAMP-LUMID Tt (replicate 1, replicate 2)
12	1	16	18.5	-	-	24.2	15 min, 15 min	n.t.
30	1	16	21	-	-	27.2	15 min, 15 min	n.d., n.d.
32	1	16	23.5	-	-	26.6	27 min, 27 min	n.t.
36	1	16	23.3	-	-	28.8	19.5 min, 19.5 min	n.d., n.d.
39	1	16	23.7	-	-	26.2	18 min, 18 min	n.t.
**HPV 18+ samples**								
Panel no	# subtypes	HPV subtype	Ct value HPV16	Ct value HPV18	Ct values (other HPV subtypes	Ct value internal control	HPV16 LAMP-LUMID Tt (replicate 1, replicate 2)	HPV18 LAMP-LUMID Tt (replicate 1, replicate 2)
5	1	18	-	20.4	-	29.1	n.d., n.d.	16.5 min, 18 min
10	1	18	-	22	-	25.9	n.d., n.d.	16.5 min, 16.5 min
17	1	18	-	23.7	-	24.5	n.t.	16.5 min, 16.5 min
20	1	18	-	25.9	-	26.2	n.t.	18 min, 21 min
37	1	18	-	29.2	-	25.9	n.t.	22.5 min, n.d.
**HPV 16/18+ samples**								
Panel no	# subtypes	HPV subtype	Ct value HPV16	Ct value HPV18	Ct values (other HPV subtypes	Ct value internal control	HPV16 LAMP-LUMID Tt (replicate 1, replicate 2)	HPV18 LAMP-LUMID Tt (replicate 1, replicate 2)
3	4	16, 18, 6, 11	21.3	27.4	34.2 (6), 34.8 (11)	29.1	15 min, 15 min	19.5 min, 21 min
**Other HPV subtypes + samples**								
Panel no	# subtypes	HPV subtype	Ct value HPV16	Ct value HPV18	Ct values (other HPV subtypes	Ct value internal control	HPV16 LAMP-LUMID Tt (replicate 1, replicate 2)	HPV18 LAMP-LUMID Tt (replicate 1, replicate 2)
2	4	73,42,82,6	-	-	24.7 (73), 19.6 (42), 31.8 (82), 30.9 (6)	28.1	n.t.	n.d., n.d.
27	1	42	-	-	19.6 (42)	26.0	n.t.	n.d., n.d.
11	1	11	-	-	25.3 (11)	29.3	n.d., n.d.	n.t.
24	3	51, 52, 11	-	-	35 (51), 37.6 (52), 20.1 (11)	28.4	n.d., n.d.	n.t.
**HPV- samples**								
Panel no	# subtypes	HPV subtype	Ct value HPV16	Ct value HPV18	Ct values (other HPV subtypes	Ct value IC	HPV16 LAMP-LUMID Tt (replicate 1, replicate 2)	HPV18 LAMP-LUMID Tt (replicate 1, replicate 2)
7	0	-	-	-	-	29.6	n.t.	n.d., n.d.
35	0	-	-	-	-	29.3	n.d., n.d.	n.t.
